# Undecaprenyl phosphate translocases confer conditional microbial fitness

**DOI:** 10.1038/s41586-022-05569-1

**Published:** 2022-11-30

**Authors:** Brandon Sit, Veerasak Srisuknimit, Emilio Bueno, Franz G. Zingl, Karthik Hullahalli, Felipe Cava, Matthew K. Waldor

**Affiliations:** 1 Division of Infectious Diseases, Brigham and Women’s Hospital, Boston, MA USA; 2 Department of Microbiology, Harvard Medical School, Boston, MA USA; 3 Laboratory for Molecular Infection Medicine Sweden (MIMS), Department of Molecular Biology, Umeå Centre for Microbial Research (UCMR), Umeå University, Umeå, Sweden; 4 Department of Immunology and Infectious Diseases, Harvard T. H. Chan School of Public Health, Boston, MA USA; 5 Howard Hughes Medical Institute, Bethesda, MD USA; 6 Present Address: Department of Biology, Massachusetts Institute of Technology, Cambridge, MA USA; 7 Present Address: Department of Biochemistry, Faculty of Science, Chulalongkorn University, Bangkok, Thailand

**Keywords:** Microbial genetics, Bacterial physiology, Pathogens, Bacteriology

## Abstract

The microbial cell wall is essential for maintenance of cell shape and resistance to external stressors^1^. The primary structural component of the cell wall is peptidoglycan, a glycopolymer with peptide crosslinks located outside of the cell membrane^1^. Peptidoglycan biosynthesis and structure are responsive to shifting environmental conditions such as pH and salinity^2–6^, but the mechanisms underlying such adaptations are incompletely understood. Precursors of peptidoglycan and other cell surface glycopolymers are synthesized in the cytoplasm and then delivered across the cell membrane bound to the recyclable lipid carrier undecaprenyl phosphate^7^ (C55-P, also known as UndP). Here we identify the DUF368-containing and DedA transmembrane protein families as candidate C55-P translocases, filling a critical gap in knowledge of the proteins required for the biogenesis of microbial cell surface polymers. Gram-negative and Gram-positive bacteria lacking their cognate DUF368-containing protein exhibited alkaline-dependent cell wall and viability defects, along with increased cell surface C55-P levels. pH-dependent synthetic genetic interactions between DUF368-containing proteins and DedA family members suggest that C55-P transporter usage is dynamic and modulated by environmental inputs. C55-P transporter activity was required by the cholera pathogen for growth and cell shape maintenance in the intestine. We propose that conditional transporter reliance provides resilience in lipid carrier recycling, bolstering microbial fitness both inside and outside the host.

## Abstract (web summary)

Members of the DUF368-containing and DedA transmembrane protein families have conditional roles in undecaprenyl phosphate translocation in Gram-negative and Gram-positive bacteria and may have a widely conserved function in the biogenesis of microbial cell surface glycopolymers.

## Main

**Fig. 1:**
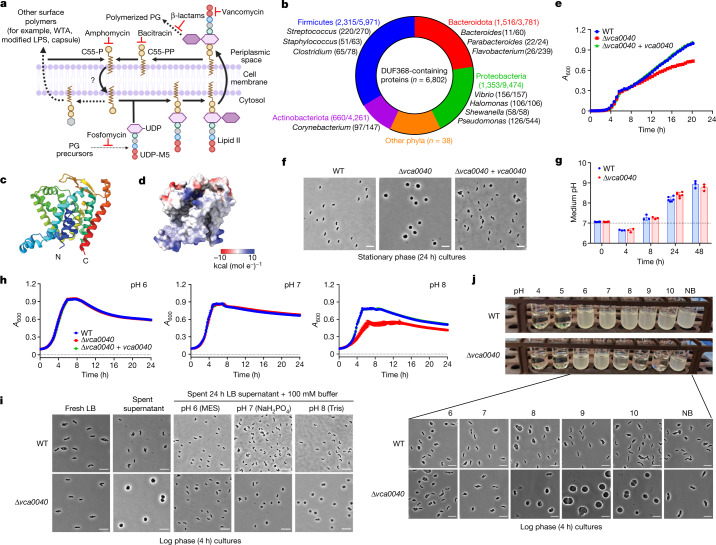
VCA0040 is required for cell shape maintenance of *V. cholerae* at alkaline pH. **a**, Usage and recycling of C55-P in bacteria. Sites of antibiotic action relevant to Fig. 3 are indicated with red bars. Dashed arrows represent multiple enzymatic and/or transport steps. The grey hexagon represents variable sugars linked to C55-P for downstream glycopolymer assembly. LPS, lipopolysaccharide; PG, peptidoglycan. **b**, Conservation of DUF368 in selected bacterial phyla (upright) and genera (italic). The coloured segments and associated labels denote selected phyla with substantial DUF368 conservation. The fraction of sequenced unique clade genomes encoding a DUF368-containing protein is indicated. **c**,**d**, Predicted ribbon (**c**) and electrostatic surface coloured (**d**) structures of VCA0040. Colour scale, −10 to 10 kcal (mol *e*^−^). **e**,**f**, Growth (**e**) and morphology (**f**) of wild-type (WT), Δ*vca0040* and Δ*vca0040* + *vca0040* (chromosomally complemented) *V*. *cholerae* in LB medium. **g**, Medium pH of *V. cholerae *overnight LB cultures. **h**, *V. cholerae* growth in M9 medium buffered to the indicated pH. **i**, The effects of buffered spent supernatant (cell-free medium after 24 h of growth at 30 °C from a 1:1,000 culture) on *V. cholerae* morphology in log phase. **j**, The effects of pH on *V. cholerae* growth (top) and morphology (bottom) during log phase in LB medium buffered with 50 mM Na_2_HPO_4_. NB, unbuffered medium. **e**,**g**,**h**, Data are mean ± s.d. from *n* = 3 cultures per strain or condition. **f**,**i**,**j**, Representative images from *n* = 3 cultures per strain or condition. Scale bars, 3 μm (**f**) and 5 μm (**i**,**j**).

Phosphorylated undecaprenyl (C55) lipids have an essential role as recyclable carrier molecules in microbial cell surface glycopolymer biogenesis^7^. During peptidoglycan biosynthesis, the sugar-linked pentapeptide subunits of peptidoglycan that are assembled in the bacterial cytoplasm are covalently linked to C55-P for transmembrane transport^7^ (Fig. 1a). After the carrier-linked peptidoglycan precursor (lipid II) is moved from the inner to outer leaflet of the membrane by MurJ^8^, subsequent incorporation of the muropeptide into polymerized peptidoglycan leaves behind C55 pyrophosphate (C55-PP). Carrier recycling is initiated by hydrolysis of C55-PP to C55-P by membrane-associated phosphatases including UppP (also known as BacA) and the PAP2-domain proteins PgpB, YbjG and LpxT^7^. Then, C55-P is presumably flipped back into the cytosolic face of the membrane to complete recycling. Preliminary structural studies have proposed that UppP may also function as a C55-P translocase^9,10^, but the protein(s) responsible for C55-P internalization have not yet been identified. C55-P recycling is a key step in the biosynthesis of peptidoglycan as well as other cell surface glycopolymers, including wall teichoic acids, certain lipopolysaccharide modifications and capsules^7^. Given its wide-ranging and critical role in cell surface maintenance, C55-P recycling is considered to be an important target for antimicrobial therapies, and naturally occurring antibiotics that inhibit this process have been identified^11^.

*Vibrio cholerae*, the Gram-negative causal agent of the diarrheal disease cholera, is exposed to dramatic changes in pH and ion concentrations as it enters, transits and exits the host gastrointestinal tract. A variety of sensing and signalling networks enable *V. cholerae* to adapt to changing environments. For example, the pathogen responds to altered salinity and pH by using a sodium motive force (SMF) instead of a proton motive force (PMF) to power protein secretion and flagellar-dependent motility, and regulate virulence gene expression^12,13^. The peptidoglycan composition of *V. cholerae* is thought to be influenced by environmental inputs, but the effects of pH on *V. cholerae* cell wall biology are unknown^14^.

## DUF368 impacts *V. cholerae* alkaline fitness

**Extended Data Fig. 1:**
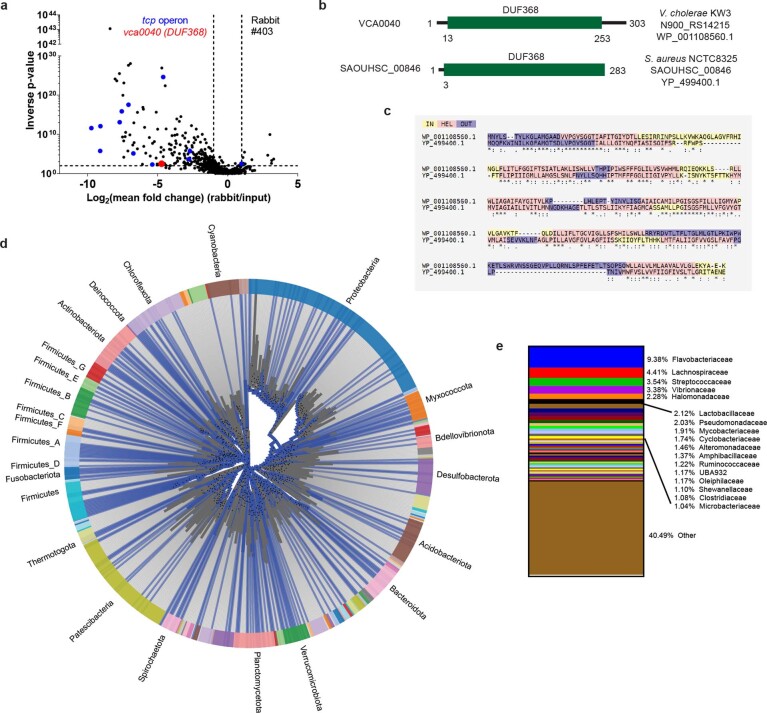
Identification and conservation of VCA0040. (**a**): Infant rabbit transposon-insertion sequencing data reproduced from Hubbard *et al*. (ref. ^15^) identifying *vca0040* (red) as a *V. cholerae* intestinal colonization determinant. The *tcp* operon, known to be critical for colonization, is highlighted in blue. One representative rabbit (#403) is shown. Inverse p-values were obtained by the Mann-Whitney U test without adjustments for multiple comparisons. Plotted thresholds are the same as Fig. 4a. (**b** and **c**): Domain structure, locus tags, accession numbers (**b**), and alignment (**c**) of *V. cholerae* and *S. aureus* DUF368-containing proteins. Alignment was performed with T-COFFEE. IN: predicted cytosol-facing sequence. HEL: predicted transmembrane helix sequence. OUT: predicted external-facing sequence. (**d** and **e**): Phylogenetic conservation of DUF368 domains (PF04018). (**d**): Family-level conservation (blue lines) grouped by phylum (colored arcs). Selected well-represented phyla are labeled. Blue lines are not scaled and do not provide information on the number or diversity of within-family DUF368 sequences. (**e**): Family-level breakdown of DUF368 conservation.

**Extended Data Fig. 2:**
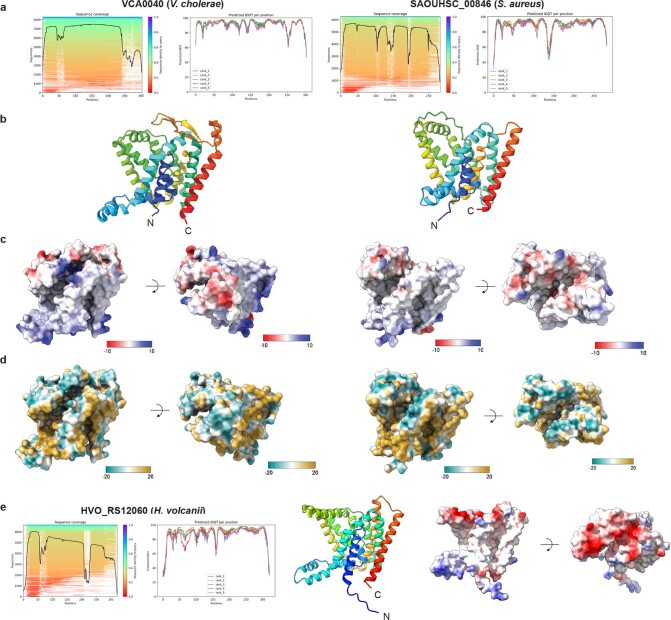
Structural modeling of DUF368-containing proteins. The *V. cholerae* (left) and *S. aureus* (right) DUF368-containing proteins were modeled using AlphaFold2 and visualized with ChimeraX. (**a**): Multiple sequence alignment (MSA) coverage (left) and pLDDT (confidence score, right) per position for each protein from AlphaFold2. (**b**): Overall ribbon structures, colored from N (violet) to C (red) termini. (**c** and **d**): Electrostatic surface potential (**c**) and lipophilicity (**d**) maps generated using default ChimeraX settings. *V.* *cholerae* panels are reproduced from Fig. 1c,d for clarity. Electrostatic colour scale: -10 to 10 kcal/(mol·e). Lipophilicity colour bar: relative molecular lipophilicity as calculated by ChimeraX. (**e**): MSA coverage, pLDDT plot, and ribbon and electrostatic surface views of the DUF368 protein from *Haloferax volcanii* (HVO_RS12060).

**Extended Data Fig. 3:**
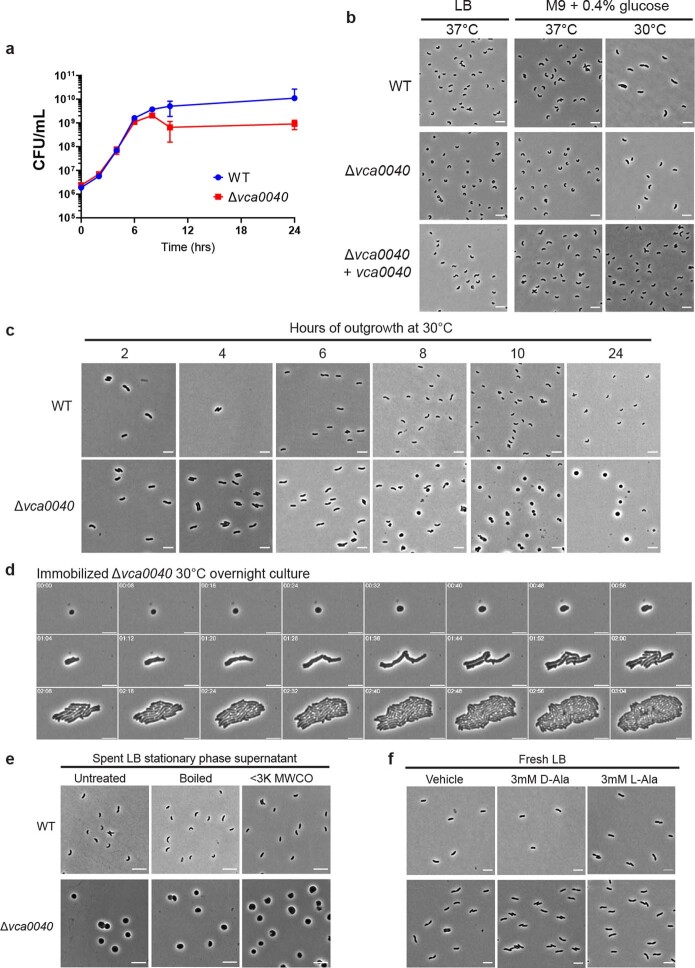
Growth and morphology of Δ*vca0040 V. cholerae*. (**a**): CFU plating time course of WT and Δ*vca0040 V. cholerae*. Onset of stationary phase occurs after 6 h. (**b**): Phase-contrast imaging of WT, Δ*vca0040* and Δ*vca0040 + vca0040 V. cholerae* in overnight cultures in the indicated conditions. (**c**): Phase-contrast imaging time course of the indicated strains grown in LB at 30 °C for the indicated amount of time. (**d**): Timelapse imaging of Δ*vca0040* cells immobilized on an agarose pad from a 30 °C overnight culture. Images were acquired over 3 h of growth. (**e**): Imaging of *V. cholerae* treated with normal, boiled (10 min) or filtered (<3K MW cutoff) cell-free spent supernatant for 4 h at 30 °C. (**f**): Imaging of *V. cholerae* treated with vehicle (DMSO) or 3 mM of D- or L-Ala in fresh LB for 4 h at 30 °C. **a**: mean ± SD of n = 3 independent cultures per strain. **b**, **c**, **e**, **f**: representative images of n = 3 independent cultures per strain/condition. **d**: representative timelapse of a single cell representative of >20 sphere-shaped bacteria across n = 3 separate fields of view from a single overnight culture. Scale bars: 3 (**b**,**c**,**f**) or 5 (**d**,**e**) µm.

A recent in vivo transposon-insertion sequencing screen for determinants of intestinal colonization in a contemporary *V. cholerae* clinical isolate identified numerous genes not previously linked to *V. cholerae* pathogenesis, including several loci of unknown function^15^ (Extended Data Fig. 1a). One of these genes, *vca0040* (N900_RS14215), was selected for further study because similar datasets^16–18^ suggested a universal requirement for this locus in *V. cholerae* intestinal colonization. VCA0040 is predicted to be a multi-pass inner membrane protein and contains the widely conserved domain of unknown function DUF368 (also known as PF04018) (Fig. 1b and Extended Data Fig. 1b–e). The predicted structural model of VCA0040 has a large putative cleft, characteristic of domains with ligand binding and/or transport activity (Fig. 1c,d and Extended Data Fig. 2). A *V. cholerae* strain lacking VCA0040 (Δ*vca0040*) exhibited a stationary phase growth defect (Fig. 1e and Extended Data Fig. 3a) that was accompanied by a distinct cell shape phenotype, where Δ*vca0040* cells became large spheres (Fig. 1f and Extended Data Fig. 3b,c). These spherical *V. cholerae* were viable and gave rise to normal rod-shaped daughter cells (Extended Data Fig. 3d).

We reasoned that a stationary phase-specific factor could be triggering the shape defect in Δ*vca0040* cells. Accordingly, exposure of exponentially growing Δ*vca0040* cells to cell-free spent supernatants from stationary phase cultures rapidly induced sphere formation (Extended Data Fig. 3e). Heat-labile and high-molecular-weight factors, as well as d-amino acids—which modulate stationary phase peptidoglycan composition^19^—were excluded as candidates (Extended Data Fig. 3e,f). In LB cultures, *V. cholerae* entry into stationary phase is accompanied by media alkalinization (Fig. 1g). Since minimal shape defects were observed in neutral buffered M9 medium (Extended Data Fig. 3b), we hypothesized that Δ*vca0040* cells were alkaline-sensitive. Buffering M9 media to different pH values revealed a growth defect at pH 8, but not at pH 6 or pH 7, and acidification of the cell-free spent supernatant ablated the sphere-induction phenotype (Fig. 1h,i). In buffered LB, Δ*vca0040* cells exhibited growth and cell shape defects at pH above 8, but not at pH values of up to 7 (Fig. 1j). Thus, the DUF368-containing protein VCA0040 is required for *V. cholerae* cell shape integrity and growth in alkaline conditions.

## Altered peptidoglycans in DUF368 mutants

**Extended Data Fig. 4:**
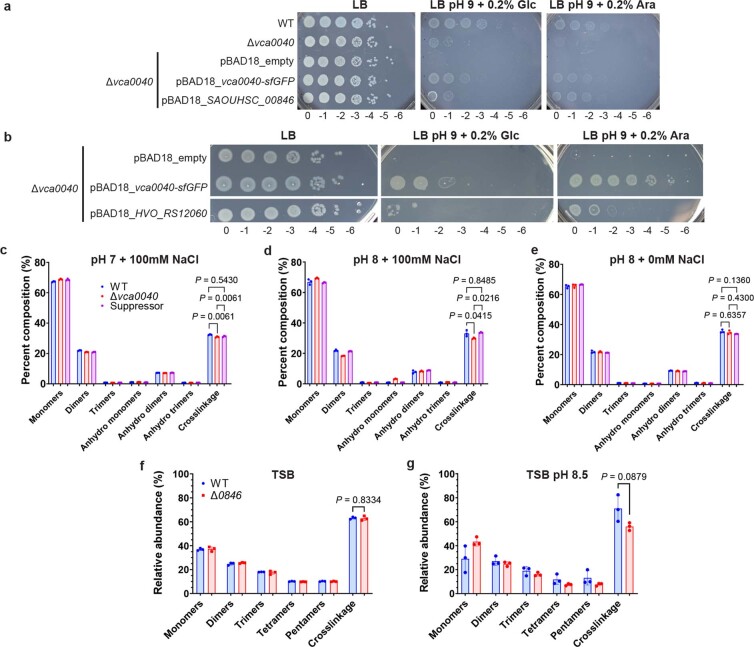
DUF368 functional conservation and effects on PG composition and crosslinking. (**a** and **b**): Alkaline growth of Δ*vca0040 V. cholerae* bearing pBAD18 vectors expressing (**a**) *SAOUHSC_00846* from *S. aureus* or (**b**) *HVO_RS12060* from *H. volcanii*. (**c**-**e**): Composition and crosslinking analysis in the indicated *V. cholerae* strains at the indicated pH in M63 media. Suppressor: Δ*vca0040/ΔsecDF1*. (**f** and **g**): Same as **c**-**e** for *S. aureus* grown in the indicated conditions. **a**, **b**: representative data from n = 3 cultures per strain/condition. **c**-**g**: mean ± SD from n = 3 cultures per strain/condition. Statistical analysis was performed with one-way ANOVAs with Tukey’s multiple comparison tests (**c**-**e**) or unpaired student’s two-tailed t-tests (**f**, **g**).

DUF368 domains are almost universally conserved across the Vibrionaceae and are present in thousands of additional Gram-negative, Gram-positive and archaeal species that inhabit a wide range of niches (Supplementary Tables 1 and 2). Heterologous expression of Gram-positive (*Staphylococcus aureus*) or archaeal (*Haloferax volcanii*) DUF368 homologues at least partially complemented the alkaline growth defect of Δ*vca0040 V. cholerae* cells (Extended Data Fig. 4a,b). Both homologues showed strong predicted structural similarity to VCA0040, suggesting that DUF368-containing protein function is conserved not only between Gram-negative and Gram-positive species, but across microbial kingdoms (Extended Data Fig. 2).

**Fig. 2:**
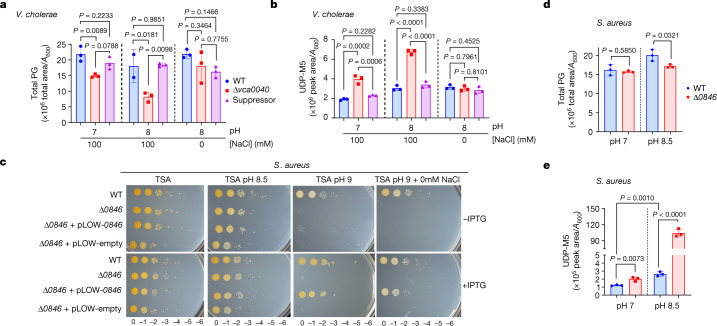
DUF368 function is conserved and necessary for peptidoglycan maintenance. **a**,**b**, Total peptidoglycan (**a**) and intracellular UDP-M5 (**b**) in *V. cholerae* grown in M63 minimal medium at the indicated pH. Suppressor, Δ*vca0040/ΔsecDF1*. **c**, Alkaline growth of Δ*SAOUHSC_00846* (Δ*0846*) *S. aureus* in the indicated conditions and with the indicated expression vectors. Representative results from *n* = 3 independent experiments per condition. **d**,**e**, Total peptidoglycan (**d**) and intracellular UDP-M5 (**e**) in *S. aureus* grown in tryptic soy broth (TSB) with 100 mM bicine at the indicated pH. **a**,**b**,**d**,**e**, *n* = 3 cultures per strain or condition; data are mean ± s.d. **a**,**b**, One-way ANOVA with Tukey’s multiple comparison test. **d**,**e**, Unpaired Student’s two-tailed *t*-test.

Since peptidoglycan is required for maintenance of bacterial cell shape, we hypothesized that DUF368 functions affect the cell wall. The Δ*vca0040* mutant had 1.5–2× less peptidoglycan than the wild type, and modest cross-linking defects with concurrent accumulation of the peptidoglycan precursor UDP-*N*-acetylmuramyl pentapeptide (UDP-M5), suggestive of a peptidoglycan biosynthesis defect upstream of cross-linking (Fig. 2a,b and Extended Data Fig. 4c–e). These phenotypes were present at neutral pH and were exacerbated by exposure to alkaline conditions. Consistent with the idea that DUF368 function is conserved, *S. aureus* lacking *SAOUHSC_00846* also had an alkaline growth defect, reduced peptidoglycan quantity, accumulation of UDP-M5 and decreased cross-linking (Fig. 2c–e and Extended Data Fig. 4f,g). We concluded from these data that DUF368-containing proteins are conditionally required to maintain peptidoglycan production and composition.

## Impaired C55-P recycling in DUF368 mutants

**Fig. 3:**
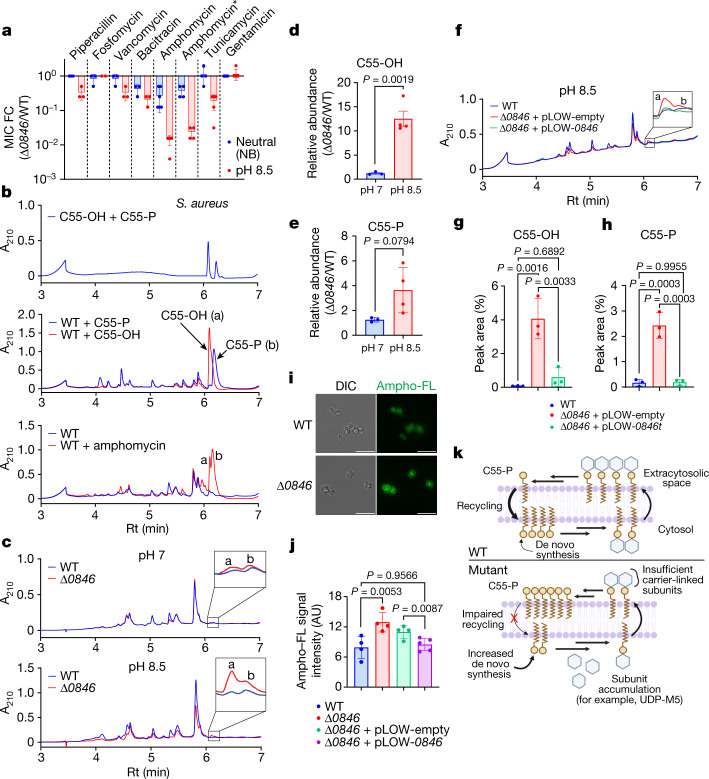
C55-P recycling is impaired in bacteria lacking DUF368-containing proteins. **a**, Fold change (FC) in MIC for Δ*SAOUHSC_00846 S. aureus* in the indicated conditions. Amphomycin*, amphomycin with 1 mM CaCl_2_. **b**, Ultra high performance liquid chromatography (UPLC) traces of purified C55-OH (a) and C55-P (b) standards (top), wild-type *S. aureus* lipid extracts spiked with each standard (middle) or wild-type *S. aureus* treated with 50 μg ml^−1^ amphomycin (bottom). Rt, retention time. **c**, UPLC traces of lipid extracts of wild-type or mutant *S. aureus* grown in TSB pH 7 (top) or pH 8.5 (bottom). **d**,**e**, Relative abundance of C55-OH (**d**) and C55-P (**e**) under same conditions as in **c**. **f**, UPLC trace of lipid extracts of complemented Δ*SAOUHSC_00846* *S. aureus* grown in TSB pH 8.5. **g**,**h**, Raw peak proportions of C55-OH (**g**) and C55-P (**h**) in each strain grown at pH 8.5. **i**, *S. aureus* stained with ampho-FL at pH 8.5. Scale bars, 5 μm. DIC, differential interference contrast image. **j**, Quantification of ampho-FL signal. Symbols represent the mean ampho-FL intensity of 1 to 8 individual bacterial clusters in independent cultures. **k**, Proposed model of disrupted C55-P recycling and the associated consequences in bacteria lacking C55-P translocase activity. **b**,**c**,**f**,**i**, Representative data from *n* = 3 cultures per strain or condition. **d**,**e**,**g**,**h**,**j**, Data are mean ± s.d. from *n* = 3–5 cultures per strain or condition. Each point is an independent culture. **d**,**e**, Unpaired student’s two-tailed *t*-test. **g**,**h**,**j**, One-way ANOVA with Tukey’s multiple comparison test.

**Extended Data Fig. 5:**
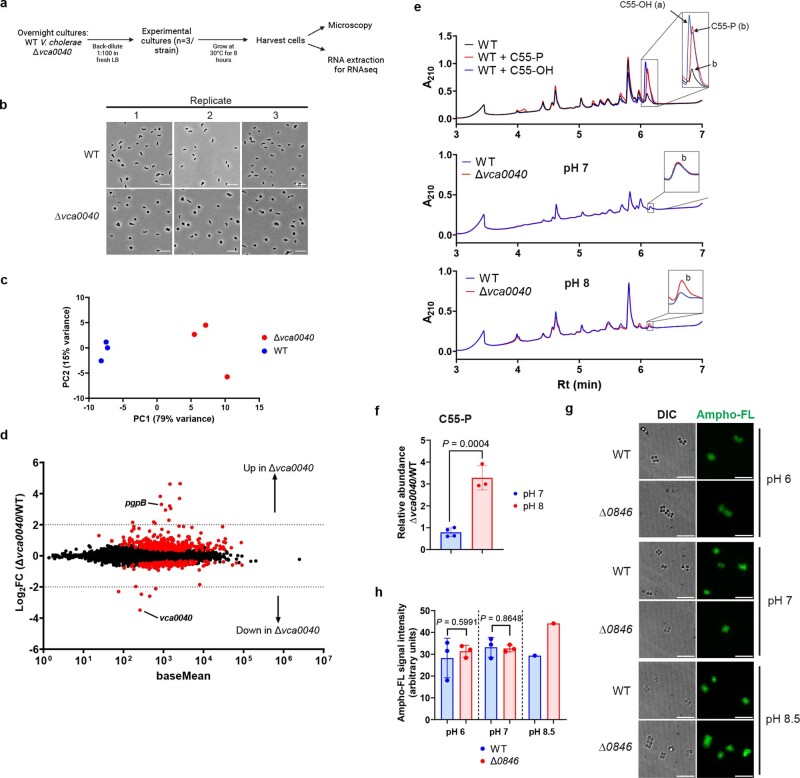
Additional characterization of bacteria lacking DUF368-containing proteins. (**a**-**d**): RNAseq of Δ*vca0040 V. cholerae*, with schematic (**a**), confirmation of sphere formation via imaging at sample collection (**b**), principal component analysis of RNAseq data (**c**), and MA plot of analyzed sequences (**d**) (n = 3 independent cultures per strain). An arbitrary fold change (FC) cutoff of 4 was implemented during RNAseq analysis, and genes with FC values beneath the p-value threshold of 0.05 are highlighted in red. *pgpB* and *vca0040* are labeled. (**e** and **f**): UPLC traces (**e**) and quantification of C55-P relative abundance (**f**) in *V. cholerae* grown at the indicated pH. (**g**): Ampho-FL-stained *S. aureus* grown at pH 6, 7, or 8.5. (**h**): Quantification of ampho-FL staining signal at different pHs as shown in **g**. Symbols represent the mean ampho-FL intensity of n = 1–8 individual bacterial clusters in independent cultures. Single pH 8.5 cultures were used as controls as a new batch of ampho-FL was used for this experiment. **e**, **g**: representative data of n = 3 cultures per strain/condition (except for n = 1 culture in pH 8.5 in **g**). **f**, **h**: n = 3 independent cultures. Statistical analysis was performed with (**d**) Wald tests in the DESeq2 pipeline with multiple comparison adjustments or (**f**, **h**) unpaired student’s two-tailed t-tests. Scale bars, 5 µm.

Two additional observations focused our investigation of DUF368 domains on cell wall assembly. First, we observed that although most DUF368-containing proteins consist primarily of one or two DUF368 domains, several microorganisms encode dual-domain proteins with DUF368–PAP2, PAP2–DUF368 or DUF368–BacA architectures (Supplementary Table 3). Second, during sphere formation in Δ*vca0040 V. cholerae*, there was an approximately tenfold induction of *pgpB*, which encodes a C55-PP phosphatase^20^ (Extended Data Fig. 5a–d and Supplementary Table 4). Despite these associations of DUF368 with C55-related processes, the Δ*vca0040* mutant exhibited only minor (up to 4×) increases in minimum inhibitory concentrations (MICs) of an array of peptidoglycan-targeting antibiotics, probably because most cell wall-acting compounds cannot penetrate the Gram-negative outer membrane (Supplementary Table 5). Accordingly, MIC screening in *S. aureus* revealed that the Δ*SAOUHSC_00846* mutant was far more sensitive (more than 64×) to amphomycin than the wild type in alkaline conditions (Fig. 3a and Supplementary Table 5). Amphomycin is a Ca^2+^-dependent lipopeptide antibiotic that specifically binds C55-P in the outer leaflet of the cytoplasmic membrane and inhibits its recycling^21–23^ (Fig. 1a). Consistent with our previous data (Fig. 2d,e), amphomycin is known to induce UDP-M5 accumulation and decrease peptidoglycan cross-linking in *S. aureus*^24^. The Δ*SAOUHSC_00846* mutant was additionally moderately sensitized to tunicamycin, which inhibits the first committed and C55-P-dependent steps of wall teichoic acid and peptidoglycan synthesis as well as bacitracin, which binds C55-PP and prevents C55-P generation on the extracytosolic face of the membrane^25,26^ (Figs. 1a and  3a). MICs for other peptidoglycan-targeting molecules such as fosfomycin were minimally changed (Fig. 3a and Supplementary Table 5). To test DUF368 function in a Gram-negative organism, we heterologously expressed *SAOUHSC_00846* or *vca0040* in the outer membrane-permeable—and thus amphomycin-sensitized—*Escherichia* *coli* strain *lptD4213*^27^ (Supplementary Table 5). Expression of either DUF368-containing protein in *lptd4213 E. coli* conferred amphomycin resistance (Supplementary Table 5). Since *E. coli* lacks an endogenous DUF368-containing protein, this result indicates that DUF368 function is sufficient for resistance to a surface C55-P-targeting antibiotic.

The peptidoglycan composition and antibiotic susceptibility data indirectly suggested that C55-P recycling was impaired in the Δ*SAOUHSC_00846* mutant, specifically in C55-P re-internalization. To further investigate this idea, we quantified C55 species in membrane lipid extracts from wild-type and Δ*SAOUHSC_00846* cultures. Amphomycin treatment of wild-type *S. aureus* led to marked accumulation of both C55-P and the Gram-positive-restricted C55-P precursor C55-OH^28^ (undecaprenol, also known as UndOH) (Fig. 3b). This phenotype was replicated in Δ*SAOUSHC_00846 S. aureus* cells grown in alkaline media, but not in neutral conditions (Fig. 3c–e) or in a complemented strain (Fig. 3f–h), providing direct evidence that C55-P homeostasis is linked to *SAOUSHC_00846*. We also observed similar alkaline-dependent increases in C55-P (but not C55-OH) in Δ*vca0040 V. cholerae* (Extended Data Fig. 5e,f). To specifically quantify C55-P in the outer leaflet of the cell membrane, we synthesized fluorescein-conjugated amphomycin (ampho-FL) and used it to label live bacterial cells^29^. Ampho-FL-labelled Δ*SAOUHSC_00846 S. aureus* exhibited increased signal relative to wild-type bacteria in alkaline media, demonstrating that surface C55-P levels are conditionally elevated in the mutant (Fig. 3i,j and Extended Data Fig. 5g,h). Together, these observations suggest that the alkaline accumulation of surface C55-P in DUF368-containing protein mutants provokes a compensatory—but ultimately insufficient—increase in C55-P synthesis, resulting in surface glycopolymer production defects and impaired growth (Fig. 3k). These findings are consistent with a model in which DUF368-containing proteins are C55-P recycling transporters that are active in alkaline conditions.

## Genetic interactions between DUF368 and DedA

**Fig. 4:**
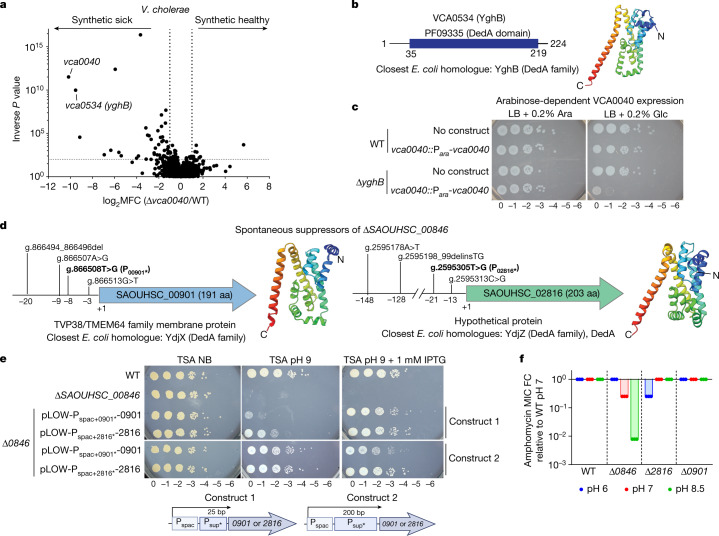
DedA family members are additional C55-P translocase candidates. **a**, A synthetic transposon screen in Δ*vca0040 V. cholerae*, showing log_2_ mean read fold changes (MFC; threshold ±2) and inverse Mann–Whitney *U* test *P*-values (threshold 100). Pooled data from two independent transposon libraries. **b**, Domain content and predicted structure of *V. cholerae* VCA0534 (also known as YghB). **c**, Growth of Δ*yghB V. cholerae vca0040* depletion strains. Ara, arabinose; Glc, glucose. **d**, Spontaneous suppressors of Δ*SAOUHSC_00846* map to two *S. aureus* DedA family proteins, with predicted structures (right) and specific promoter mutations selected for validation (bold). **e**, Rescue of Δ*SAOUHSC_00846* by expression of *SAOUHSC_00901* (*0901*) or *SAOUHSC_2816* (*2816*) from hybrid promoters containing the IPTG-inducible promoter P_spac_ linked to 25-bp (construct 1) or 200-bp (construct 2) native promoter stretches with the indicated suppressor mutations (*). **f**, Fold change in amphomycin MIC for wild-type and mutant *S. aureus* relative to the MIC for the wild type at pH 7 (150 μg ml^−1^). **c**,**e**, Representative results from *n* = 3 independent experiments per condition. **f**, Data are mean ± s.d. from *n* = 3 cultures per strain or condition.

**Extended Data Fig. 6:**
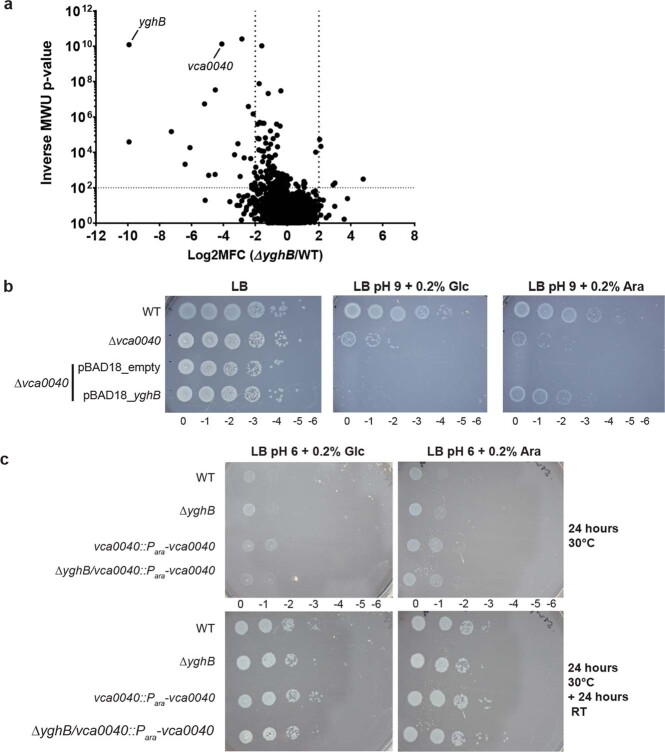
Genetic interactions between DedA and DUF368 proteins. (**a**): Synthetic transposon-insertion screen in Δ*yghB V. cholerae*. P-values were obtained by the Mann-Whitney U (MWU) test without adjustment for multiple comparisons. MFC: mean fold change in read coverage between Δ*yghB* and WT. (**b**): Growth of Δ*vca0040 V. cholerae* expressing vectors with arabinose-inducible genes on regular LB (left), LB pH 9 (middle) and LB pH 9 + arabinose (right) plates. (**c**): Growth of *V. cholerae* lacking one or both of *vca0040* and/or *yghB* on LB pH 6 plates. LB was buffered with 100 mM MES and pH-adjusted with HCl. **a**: pooled data from n = 2 independent transposon libraries. **b**, **c**: representative images from n = 3 cultures per strain/condition.

As C55-P recycling is thought to be an essential function and DUF368 mutants were conditionally viable, we next carried out a synthetic transposon screen to define the genetic network of *vca0040*. Many of the identified synthetic sick or lethal interactions were related to cell envelope homeostasis (Fig. 4a and Supplementary Table 6). The strongest interaction of *vca0040* was with *N900_RS16280* (also known as *vca0534*), which encodes a homologue of the *E. coli* DedA family member YghB (Fig. 4b). *V. cholerae* express two other DedA proteins, but these were not hits in this screen. DedA family transmembrane proteins (also known as PF09335 (SNARE-associated Golgi protein)) are conserved in all three domains of life, are generally required for cell envelope homeostasis, and are suspected to mediate PMF-dependent transport, but their specific substrates are unknown^30,31^. Similar to DUF368, although most DedA-containing sequences encode only that domain, there are many instances of domain architectures in which DedA is fused to the C55-PP phosphatase domain PAP2 (Supplementary Table 3). The synthetic lethality of *vca0040* and *yghB* was validated by genetic depletion and reciprocal synthetic transposon screening (Fig. 4c, Extended Data Fig. 6a and Supplementary Table 7), and overexpression of YghB rescued the alkaline defect of Δ*vca0040 V. cholerae* (Extended Data Fig. 6b).

Interactions between DUF368 and DedA proteins were also observed in *S. aureus*. In alkaline-enriched spontaneous suppressors of Δ*SAOUHSC_00846 S. aureus*, we found frequent promoter (and no coding region) mutations in the genes encoding the two *S. aureus* DedA proteins (SAOUHSC_00901 and SAOUHSC_02816) (Fig. 4d). Incorporating these mutations into an isopropyl β-d-1-thiogalactopyranoside (IPTG)-inducible system expressing either DedA protein rescued Δ*SAOUHSC_00846* even without induction, suggesting that the isolated mutations increase gene expression and compensate for the absence of *SAOUHSC_00846*, as in *V. cholerae* (Fig. 4e). These genetic studies suggest that DedA family members are also required for C55-P translocation, in agreement with recent reports that eukaryotic DedA proteins are associated with lipid transport and bacterial DedA mutants are defective in C55 carrier-dependent lipopolysaccharide modifications^31–33^.

We hypothesized that similar to DUF368-containing proteins, DedA family members function conditionally. Although deletion of *SAOUHSC_00901* did not affect amphomycin MICs, the Δ*SAOUHSC_02816* mutant exhibited moderate acid-dependent amphomycin sensitivity, strengthening the association of DedA family members with C55-P translocation and suggesting that they function at lower pH ranges than their DUF368-containing counterparts (Fig. 4f). The sensitivity of Δ*SAOUHSC_02816* to other antibiotics was unaltered at any pH, highlighting the specificity of conditional amphomycin sensitization (Supplementary Table 5). The concept of pH-dependent translocase contributions is also supported by the alkaline amphomycin sensitivity of *lptd4213 E. coli*, which has only DedA paralogues and no cognate DUF368-containing protein (Supplementary Table 5). A strain lacking *yghB* and *vca0040* was viable at acidic pH, suggesting at least one other *V. cholerae* protein can carry out C55-P translocation, or that C55-P may spontaneously traverse the membrane in acidic conditions (Extended Data Fig. 6c).

## Na^+^ shapes C55-P translocase biology

**Extended Data Fig. 7:**
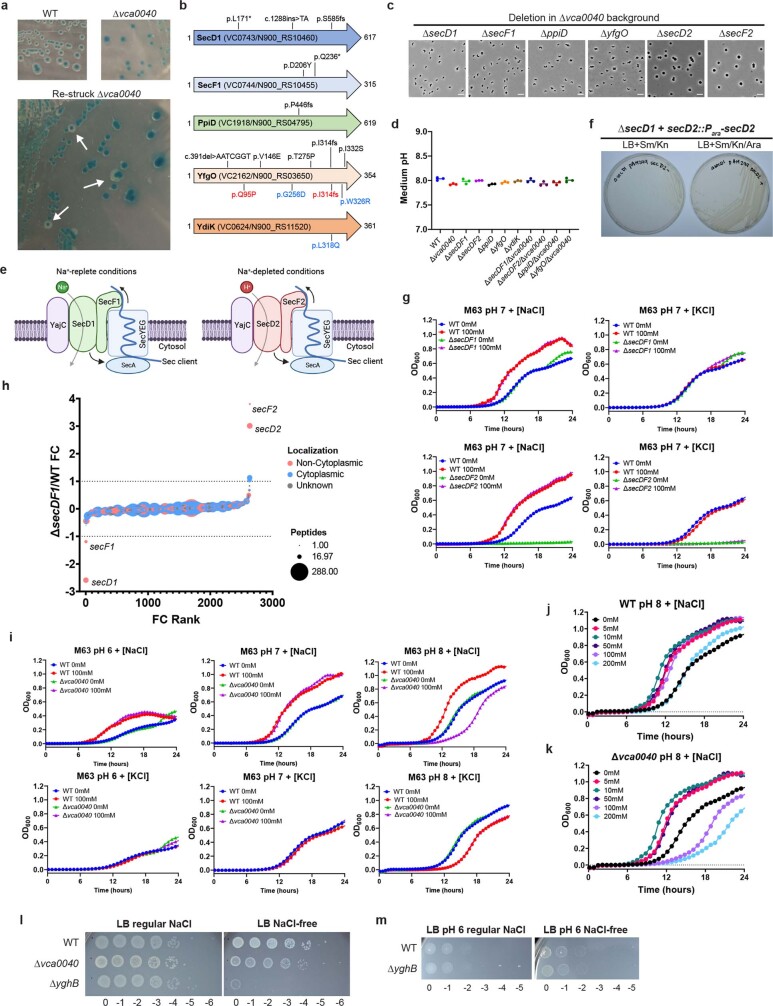
Contribution of Na^+^ to Δ*vca0040* alkaline susceptibility. (**a**): Representative colonies of WT, Δ*vca0040*, and suppressor *V. cholerae* on LB+X-gal plates. White arrows indicate suppressors. (**b**): Spontaneous suppressor mutations of Δ*vca0040 V. cholerae*. SecD1, SecF1 and PpiD are part of the *V. cholerae* protein secretion machinery. Colored text indicates suppressors from a secondary screen in Δ*secD2/*Δ*vca0040* (blue) or Δ*secF2*Δ*vca0040* (red) *V. cholerae*. Asterisk: stop codon. (**c**): Phase-contrast imaging of overnight 30 °C LB cultures of the indicated strains in the Δ*vca0040* background. Scale bars, 3 μm. (**d**): Overnight 30 °C LB culture pH of the indicated strains. (**e**): SecDF1 and SecDF2 functions in *V. cholerae*. Possible SecDF functions: coupling ion import to SecA ATPase stimulation (bottom arrow) or Sec substrate guidance into the periplasm (top arrow). (**f**): Synthetic lethality of *secD2* in Δ*secD1 V. cholerae* using the pAM299 suicide vector for Ara-dependent *secD2* expression. (**g**): Growth of the indicated strains in M63 media with added NaCl or KCl. (**h**): Waterfall plot comparing Δ*secDF1* to WT *V. cholerae* proteome ranked by protein fold change (FC). (**i**): Growth of Δ*vca0040 V. cholerae* in [Na^+^]-varying (top) or [K^+^]-varying (bottom) M63 media at pH 6, 7 or 8. (**j** and **k**): Growth of WT (**j**) or Δ*vca0040* (**k**) *V. cholerae* in M63 pH 8 with a range of [NaCl]. 100mM NaCl curves from **i** are replicated in **j** and **k** for comparison. (**l** and **m**): Growth of the indicated *V. cholerae* strains on non-buffered (**l**) or pH 6 (**m**) LB with regular (~170 mM) or no added NaCl. **a**: representative image of n = 11 independent suppressor strains. **c**, **f**, **l**, **m**: representative data of n = 3 cultures per strain/condition. **d**: means ± SD of n = 3 cultures per strain. **g**, **i**, **j**, **k**: mean ± SD of at least 3 technical replicates of single cultures (representative of n = 3 cultures per strain/condition). **h**: pooled data comparing n = 4 independent cultures of each strain.

Analyses of spontaneous suppressors of Δ*vca0040 V. cholerae*, which have distinctive colony morphology (Extended Data Fig. 7a) revealed that the requirement of VCA0040 for *V. cholerae* fitness is controlled by Na^+^ concentration in addition to pH. Whole-genome sequencing of suppressor colonies lacking cell shape defects revealed four suppressor genes, three of which (*secD1, secF1* and *ppiD*) function in Sec-mediated protein secretion (Extended Data Fig. 7b). The fourth—*yfgO*—encodes a protein of unknown function from the AI-2E transporter family, members of which have recently been implicated in peptidoglycan biosynthesis and Na^+^/H^+^ antiport^34,35^. Deletions of suppressor genes rescued the shape and peptidoglycan defects of Δ*vca0040 V. cholerae*, even though stationary culture pH was alkaline in all suppressors (Fig. 2a,b and Extended Data Fig. 7c,d). In *V. cholerae*, SecD–SecF is duplicated as SecD1–SecF1 and SecD2–SecF2, ancillary complexes that differentially couple SecYEG activity to the SMF or PMF, respectively^36^ (Extended Data Fig. 7e). The activity of at least one SecD–SecF pair was required for *V. cholerae* viability, and deletion of *secD2* and *secF2* did not ameliorate the stationary phase cell shape defect of Δ*vca0040* (Extended Data Fig. 7c,f,g). We initially hypothesized that altered Sec substrate specificity could explain the identification of SecD1 and SecF1 as suppressors. However, the Δ*secDF1 V. cholerae* proteome was unchanged from that of wild-type cells, aside from the expected increases in SecD2 and SecF2, suggesting that the suppressive effects of *secD1* and *secF1* loss were attributable to reduced Na^+^ flux (Extended Data Fig. 7h and Supplementary Table 8). Indeed, increasing Na^+^ but not K^+^ concentration induced alkaline growth and peptidoglycan defects in Δ*vca0040 V. cholerae* (Fig. 2a,b and Extended Data Fig. 7i–k). The requirement of increased Na^+^ concentration for the alkaline defect of DUF368-deficient *V. cholerae* appeared to be species-specific, as Δ*SAOUHSC_00846 S. aureus*, although alkaline-sensitive, was not similarly rescued by Na^+^ depletion (Fig. 2c). Of note, unlike Δ*vca0040*, a Δ*yghB V. cholerae* mutant was not able to grow without Na^+^ at pH values above 6 (Extended Data Fig. 7l,m), suggesting that the presence of Na^+^ divergently affects distinct C55-P translocases. Together, these data illustrate that multiple environmental inputs can control C55-P translocase requirements and potentially function in different microorganisms.

## DUF368 enables *V. cholerae* pathogenesis

**Fig. 5:**
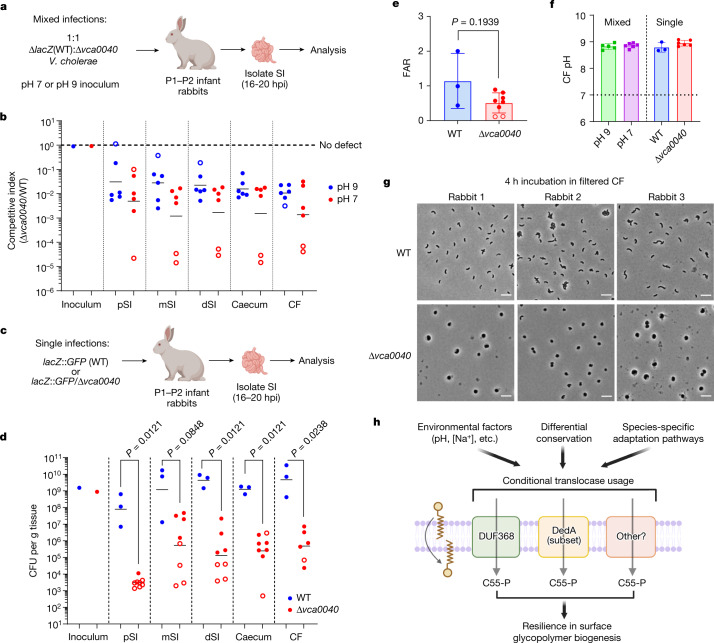
DUF368 is required for *V. cholerae* pathogenesis. **a**,**b**, Schematic (**a**) and intestinal competitive indices (**b**) in mixed-infection models (*n* = 6 rabbits in each group). SI, small intestine. **c**–**e**, Schematic (**c**), number of intestinal colony-forming units (CFU) (**d**) and fluid accumulation ratio (FAR) (**e**) in single infections (*n* = 3 rabbits (wild type) and *n* = 8 rabbits (Δ*vca0040*)). CF, caecal fluid; pSI, proximal small intestine; mSI, medial small intestine; dSI, distal small intestine. Open circles indicate rabbits with limit of detection (LOD) measurements where the true CFU burden is at least (for upper LOD circles) or at most (for lower LOD circles) the plotted value. Note two Δ*vca0040* animals had insufficient caecal fluid accumulation for FAR calculation and were assigned LOD values (arbitrary 25 μl volume). **b**,**d**, Data are geometric mean. **d**, Two-tailed Mann–Whitney *U* tests. **e**, Data are mean ± s.d. analysed with an unpaired Student’s two-tailed *t*-test. **f**, pH of caecal fluid from infected animals. Data are mean ± s.d. **g**, Incubation of laboratory-grown *V. cholerae* with filtered caecal fluid samples from three different rabbits. Representative images from *n* = 3 ex vivo incubations with independent caecal fluid samples. Scale bars, 3 μm. **h**, Proposed model for microbial C55-P translocation and its integrated control by environmental inputs, the presence of other translocases, and non-translocase-related environmental adaptation mechanisms. Source data

**Extended Data Fig. 8:**
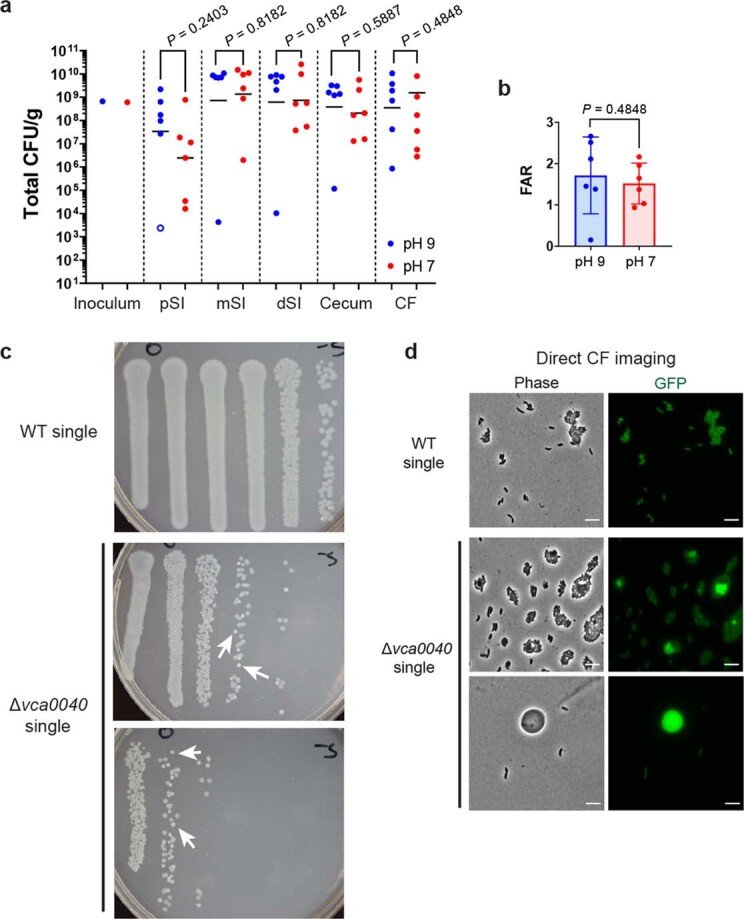
Supplemental data for infant rabbit infections. (**a**): Total CFU in mixed infections with differing inoculum pH (n = 6 each for pH 7 and 9). (**b**): Fluid accumulation ratio (FAR) in mixed infections with differing inoculum pH. (**c**): Representative small intestinal CFU plates from single infections showing transmission of WT *V. cholerae* (greyish translucent colonies) from WT-infected rabbits to Δ*vca0040*-infected rabbits in the same litter. Note that Δ*vca0040* colonies (white arrows) are lighter and smaller than WT colonies, making them visually distinguishable and countable. (**d**): Direct imaging of GFP+ *V. cholerae* in single infection CF samples. Large GFP-bright spheres are likely Δ*vca0040* cells in the presence of transmitted, rod-shaped WT cells. Scale bars, 3 μm. **a**: geometric means analyzed with two-tailed Mann-Whitney U tests. **b**: mean ± SD analyzed with an unpaired student’s two-tailed t-test. **c**, **d**: representative images from n = 3 WT and n = 7 Δ*vca0040* singly-infected rabbits. Source data

**Extended Data Fig. 9:**
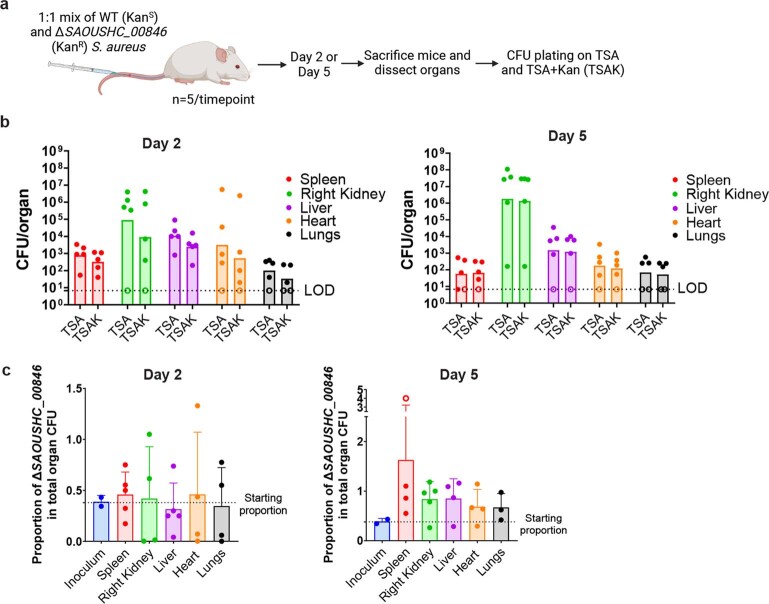
Intravenous (IV) infections of mice with *S. aureus*. (**a**): Schematic of IV infections and sample harvesting workflow. TSA counts indicate total *S. aureus* CFU burden, whereas TSAK counts indicate Δ*SAOUHSC_00846* burden. N = 5 mice were used per timepoint (n = 10 total). (**b**): Raw CFU counts from TSA and TSA + kanamycin (TSAK) plates. In the absence of a competitive defect, the TSAK counts should be 50% of those on the TSA plates (and thus are very close together on the log_10_-plotted graph). (**c**): Ratio of Δ*SAOUHSC_00846* (KanR) colony-forming units (CFU) to total *S. aureus* CFU at Day 2 (left) and Day 5 (right) post-infection. The dotted line indicates the mean starting proportion from n = 2 technical replicate plates of the inoculum. Open circles represent the limit of detection (LOD). Note that samples with CFU counts below the LOD were not plotted. **b**: geometric means. **c**: means ± SD. Source data

We used an infant (postnatal day (P)1–P2) rabbit model that mimics severe human cholera^37^ to investigate the role of VCA0040 in pathogenesis. Typically, this model uses alkaline inocula to buffer the acidic stomach environment. To exclude inoculum effects, we carried out mixed infections with inocula comprising a 1:1 mix of wild-type and Δ*vca0040 V. cholerae* at pH 7 or pH 9 (Fig. 5a). Regardless of inoculum pH, the *Δvca0040* mutant had a profound competitive defect (100–1,000x) compared with the wild type (Fig. 5b). The pH of the inoculum did not affect total colonization or accumulation of diarrhea-like caecal fluid, the primary marker of disease in this model (Extended Data Fig. 8a,b). In single infections with fluorescent wild-type or Δ*vca0040 V. cholerae*, the mutant exhibited severe colonization defects (around 1,000-fold) in all intestinal segments (Fig. 5c,d). These values overestimate the true colonization of the mutant, because rabbits from each group were co-housed to exclude litter effects, enabling substantial transmission from wild-type-infected to Δ*vca0040*-infected rabbits (Extended Data Fig. 8c). Transmission probably explains why caecal fluid accumulation in Δ*vca0040*-infected rabbits was lower than in wild-type rabbits but did not reach statistical significance (Fig. 5e). Caecal fluid collected from both mixed and singly infected animals was strongly alkaline (pH 8.5–9) (Fig. 5f). Imaging of caecal fluid samples from Δ*vca0040*-infected rabbits revealed large, fluorescent, spherical cells similar to those observed in alkaline cultures of the Δ*vca0040* mutant (Extended Data Fig. 8d). Similarly, when freshly grown rod-shaped wild-type or Δ*vca0040 V. cholerae* were incubated in cell-free caecal fluid samples. Δ*vca0040*—but not wild-type—cells became spherical when exposed to caecal fluid, suggesting that VCA0040 function is needed to maintain *V. cholerae* shape in the alkaline in vivo milieu (Fig. 5g). An infection defect was not detected for Δ*SAOUHSC_00846 S. aureus* in a mouse intravenous infection–organ abscess model, in which the pathogen is unlikely to encounter alkaline environments (Extended Data Fig. 9).

## Discussion

We propose that DUF368-containing and DedA family proteins are C55-P translocases that provide functional resiliency in this crucial step of envelope maintenance (Fig. 5h). The identification of C55-P transport proteins provisionally fills a major gap in the C55 lipid carrier recycling pathway. As our genetic and phenotypic data do not definitively exclude transport-independent activities, biochemical and structural studies are required to confirm whether these two families indeed carry out this function. The apparent pH and ion dependency of the contributions of these candidate translocases to fitness suggests that these proteins could be energetically controlled, since a major effect of pH on the microbial cell is the governance of transmembrane ionic gradients. For example, DUF368-containing proteins appear to contribute most in alkaline conditions where the PMF is low^38^, suggesting that if C55-P translocation is energy-dependent, DUF368-mediated transport may depend on the SMF or another non-PMF energy gradient. It is also possible that protonation states of C55-P, which are probably pH-dependent, determine the maximally active translocase or potential contribution of spontaneous C55-P scrambling. Characterizing the potential energetic reliance and directionality of multiple C55-P translocases will be an important topic of future study.

**Extended Data Fig. 10:**
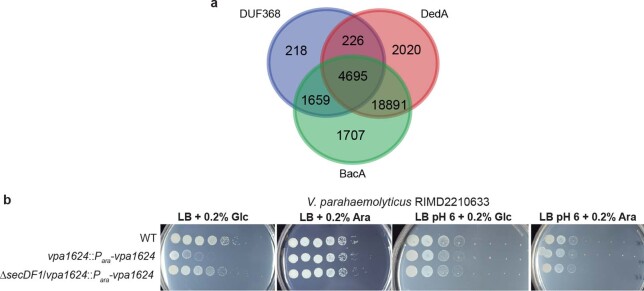
Distribution of putative C55-P translocases in bacteria and genetic analysis of a DUF368 protein in *V. parahaemolyticus*. (**a**): Venn diagram of Annotree species-level outputs for PFAM queries PF04018 (DUF368), PF09335 (DedA), and PF02673 (BacA/UppP). Out of 30,255 bacterial species in the Annotree database, 29,416 (97.2%) are predicted to contain at least one of the three proteins. A list of species in each Venn diagram segment is listed in Supplementary Table 9. This is likely also an overestimate of the number of species lacking any of these three domains due to sequence divergence and potentially inadequate PFAM annotation. For example, the obligate intracellular pathogen *Rickettsia rickettsii* (GCA_000018225.1) is in the triply-absent group, but upon manual curation is known to have peptidoglycan and has an annotated DedA coding sequence, perhaps reflecting a PFAM annotation lag. Interestingly, however, *Mycoplasma* species, which do not have lipopolysaccharide or peptidoglycan (and thus may not be dependent on C55-P translocation) appear to be genuinely triply-absent except for a select few members with only a DUF368-containing protein. (**b**): Growth of *V. parahaemolyticus* lacking the DUF368-containing protein VPA1624 at neutral and acidic pH. VPA1624 genetically interacts with the *V. parahaemolyticus secDF1* loci, as deletion of these suppressors originally identified in *V. cholerae* rescues the *vpa1624*-depleted strain. Ara: arabinose, Glc: glucose. Representative data of n = 3 independent cultures per strain/condition.

The conditionality of DUF368-containing and DedA family proteins, and perhaps additional as-yet-unidentified translocases, could support C55-P flux in diverse microbial niches (Fig. 5h). The sensitivity of Δ*vca0040 V. cholerae* to increased sodium ion concentration and the apparent enrichment of DUF368 in other known halophiles such as *S. aureus* and the archaeal class Halobacteria (including *H. volcanii*) suggests that regardless of their energetic input, DUF368-containing proteins may facilitate microbial adaptation to high-salt environments (Supplementary Table 1). Specifically, the sodium and pH-dependence of the contribution of VCA0040 to *V. cholerae* physiology may reflect the robust capacity of the cholera pathogen to colonize the human small intestine, where alkaline conditions and millimolar-scale sodium concentrations are likely to occur^39^. Alongside the variable conservation of redundant translocases (Extended Data Fig. 10a and Supplementary Table 9), additional inputs and adaptation mechanisms likely also shape how C55-P recycling contributes to microbial fitness. For example, although *vca0040* is required only in alkaline conditions in *V. cholerae*, this gene is essential in the related pathogen *Vibrio parahaemolyticus* even at neutral pH, despite the conservation of *yghB* and suppressor loci, suggesting that the pH setpoint at which DUF368 family activity is dominant is not universal^40^ (Extended Data Fig. 10b). Although DUF368-containing proteins—which have been renamed polyprenyl phosphate transporter (PopT) proteins in concurrently reported work^41^—are restricted to bacteria and archaea, DedA family members are widely present in eukaryotes, including humans. Thus, our findings may affect the understanding of polyprenyl phosphate (for example, dolichol phosphate) translocation in all kingdoms of life.

## Methods

### Bacterial strains, media and growth conditions

All *V. cholerae* strains used in this study are derivatives of HaitiWT, a spontaneous streptomycin-resistant variant of a clinical isolate from the 2010 Haiti cholera epidemic^42^. All *S. aureus* strains used in this study are derivatives of HG003 (itself a derivative of NCTC8325). Strain and plasmid information is listed in Supplementary Table 10. All constructed strains were verified by Sanger sequencing of the targeted locus (Genewiz).

The following media were used in this study: lysogeny broth (LB) Miller (10 g l^−1^ NaCl) (BD Biosciences, USA), M9 minimal media (12.8 g l^−1^ Na_2_HPO_4_·7H_2_O, 3 g l^−1^ KH_2_PO_4_, 0.5 g l^−1^ NaCl, 1 g l^−1^ NH_4_Cl, 1 mM MgSO_4_ and 10 µM CaCl_2_) (lab-made), M63 minimal media (2 g l^−1^ (NH_4_)_2_SO_4_, 13.6 g l^−1^ KH_2_PO_4_, 0.5 mg l^−1^ FeSO_4_·7H_2_O and 1 mM MgSO_4_) (US Biologicals), TSB and tryptic soy agar (TSA) (BD Biosciences). M9 was supplemented with 0.4% glucose and pH-adjusted with NaOH. M63 was supplemented with 2% glucose and pH-adjusted with KOH. In general, LB was buffered with 50 mM Na_2_HPO_4_ or 100 mM Tris and pH-adjusted with NaOH or HCl, and TSB was buffered with 100 mM bicine. Variations in buffering are noted in figure legends. Plates were used at 1.5% final agar concentration. Where necessary, the following antibiotics or supplements were used: streptomycin (200 μg ml^−1^), carbenicillin (50 µg ml^−1^), kanamycin (50 µg ml^−1^), neomycin (50 ug ml^−1^), targocil (10 μg ml^−1^), erythromycin (10 μg ml^−1^), diaminopimelic acid (DAP, 0.3 mM), 5-bromo-4-chloro-3-indolyl β-d-galactopyranoside (X-gal, 60 µg ml^−1^), arabinose (0.2%) or IPTG (1 mM).

For routine culture, *V. cholerae* were grown in non-buffered LB at 37 °C and *S. aureus* were grown in non-buffered TSB at 37 °C. Cultures for spent supernatant analysis in *V. cholerae* were obtained by subculturing 37 °C-grown overnight cultures 1:1,000 in fresh media and growing for 24 h at 30 °C. To collect spent supernatants, cultures were centrifuged for 10 min at 5,000*g* at 4 °C. Supernatants were transferred to a new tube, re-centrifuged, and sterile filtered with 0.22 µm syringe filters into new tubes. Supernatants were stored at 4 °C for future use. Cultures used for pH quantification were centrifuged for 10 min at 5,000*g* before measurement. All pH measurements, including for media and buffer formulation, were performed with a pH meter (Thermo ORION) freshly calibrated to two appropriate pH standards from 4, 7 and 10.

### Bioinformatic methods

Information on the loci, genomes, and accession numbers for genes and proteins from this study is available in Supplementary Table 10.

#### Genomic locus correspondence in *V. cholerae*

During our study, which mainly used a contemporary pandemic strain of *V. cholerae* (HaitiWT, also known as KW3, assembly accession GCA_001318185.1), we noted several annotation inconsistencies with genes ostensibly conserved in the reference shotgun assembly (GCA_000006745.1) of N16961 *V. cholerae*. Compared to newer long-read assemblies (GCA_003063785.1 and GCA_900205735.1), the original assembly of N16961 has ~50 more ORFs annotated as pseudogenes due to frameshifts that are not included in coding sequence tables. Three of these were directly related to our study: (1) *lacZ*, a widely used reporter gene (VC2338), (2) *secF2* (VCA0692), and (3) *yghB* (VCA0534). *lacZ* is known to be functional in N16961, and re-sequencing of our laboratory N16961 stock and examination of newer N16961 assemblies confirmed that *secF2* and *yghB* are in fact intact in this strain and lack the predicted frameshift. The ‘pseudogene’ annotation of *secF2* led to its recent usage as a safe-harbour locus for genome editing in *V. cholerae*^43^, and caution is probably required for this method. To resolve the issues of difference between strain genomes, a batch BLAST dictionary assigning putative VC locus tags (the conventional *V. cholerae* gene naming system) to annotated HaitiWT loci is given in Supplementary Table 11.

#### Phylogenetic and domain architecture analysis

To identify sequences with annotated DUF368 domains, Annotree^44^ was used with the Pfam identifier PF04018 at a cut-off e-value of 1x10^−30^. The results from this search are included in Supplementary Table 1. Taxa with ‘_X’ names were manually collated into a single group for ease of analysis. In a related effort, to identify annotation-independent homologues of *vca0040*, we used a HMMER search with the VCA0040 sequence from wild-type *V. cholerae* (Supplementary Table 2). To analyse the relative distributions of PF04018, PF09335 (DedA), and PF02673 (BacA), we used Annotree at a cut-off e-value of 1 × 10^−15^. Differential species lists with all possible conservation combinations were visualized with a Venn diagram generator (https://bioinformatics.psb.ugent.be/webtools/Venn/) and are provided in Supplementary Table 9.

To assess domain architecture, we used InterProScan (https://www.ebi.ac.uk/interpro) with DUF368 (PF04018) or DedA (PF09335) as queries and manually examined identified domain structures. Sequences with domain architectures relevant to this study were exported and collated in Supplementary Table 3.

#### Structural modelling

Structural prediction of DUF368 and DedA family proteins in this study was performed with AlphaFold2 on the CoLabFold publicly accessible interface^45^. Sequences were modelled as monomers using mmseqs2 for multiple sequence alignment. Structures were ranked by pLDDT and the top-ranked structures were visualized with ChimeraX (UCSF).

### Cloning, vectors and strain construction

#### *V. cholerae*

Cloning of expression vectors and deletion plasmids was performed by standard isothermal assembly techniques with 25-bp overhangs on each fragment (HiFi DNA Assembly Kit, NEB). *V. cholerae* mutants were generated by allelic exchange as previously described with the suicide vector pCVD442 bearing 500–700 bp upstream and downstream homology arms of the targeted locus^46,47^. Either SM10λpir or MFDλpir *E. coli* (a DAP auxotroph) were used as the donor strain with identical conjugation conditions apart from DAP supplementation. Single crossover transconjugants were isolated by selective plating on LB + streptomycin/carbenicillin and passaged in 10% sucrose overnight at room temperature to select for double crossover events. Cells were plated on LB + streptomycin, re-patched onto LB + streptomycin and LB + streptomycin/carbenicillin, and streptomycin- and carbenicillin-resistant colonies were screened by colony PCR for successful deletion. For deletions involving *vca0040*, *vca0040* was always deleted last and at least two clones per strain were stocked and verified for the correct phenotype to guard against spontaneous suppressor formation. For native *vca0040* expression from the ectopic chromosomal site, the full coding sequence along with 350 bp upstream was cloned to include the native promoter and inserted at a known neutral genomic location in *V. cholerae*^48^. For expression of *SAOUHSC_00846* from the *vca0040* locus in Δ*vca0040 V. cholerae*, we used allelic exchange to replace the *vca0040* coding sequence with a *SAOUHSC_00846* sequence codon-optimized for *V. cholerae*. For depletion of *yghB* from Δ*vca0040 V. cholerae*, we used pAM299, a suicide vector which replaces the native allele of targeted gene with an arabinose-inducible copy^49^. For overexpression studies, we used pBAD18 for plasmid-based arabinose induction^50^.

#### *S. aureus*

*S. aureus* Δ*SAOUHSC_00846* was generated with a one-step allelic exchange using the pTarKO plasmid bearing 1,000 bp upstream and downstream homology arms of the targeted locus flanking a kanamycin cassette as previously described^51^. In brief, the assembled pTarKO plasmid was electroporated into electrocompetent *S. aureus* RN4220 tarO_off_ and selected for double crossovers on TSB with kanamycin, neomycin and targocil. The kanamycin insertion in *SAOUHSC_00846* was then transduced to *S. aureus* HG003 with phage phi85. An identical protocol was used to generate Δ*SAOUHSC_02816*. For Δ*SAOUHSC_0901*, the strain NE1150, which carries an erythromycin-resistance-marked transposon insertion in *SAOUHSC_00901*^52^, was used as the donor for transduction into HG003. For overexpression studies, we used pLOW for plasmid-based IPTG induction^53^. Assembled plasmids were electroporated into RN4220 wild type and transduced to *S. aureus* HG003 ∆*SAOUHSC_00846* with phage phi85.

#### *E. coli lptD4213* arabinose suppressor selection

*E. coli* MC4100 and its derivative *lptD4213* are sensitive to arabinose due to the araD139 mutation. To enable the use of the pBAD arabinose expression system, spontaneous arabinose-resistant mutants of MC4100 *lptd4213* were isolated as previously described^54^. In brief, overnight cultures of *lptD4213 E. coli* were plated (~10 µl) on LB + 0.4% arabinose plates and incubated at 37 °C. Putative suppressor colonies were purified on a new LB + 0.4% arabinose plate. Suppressor colonies were then screened for growth on LB + 0.4% arabinose and no growth on M9 minimal agar + 0.2% arabinose, in order to select for strains that are resistant to arabinose, but unable to use arabinose as carbon source (that is, *ara*^*−/r*^, but not *ara*^*+*^ colonies). To transform *E. coli* with various vectors, competent cells were heat shocked according to a standard protocol and selected on LB supplemented with the appropriate antibiotic(s).

#### *V. parahaemolyticus*

*V. parahaemolyticus* RIMD2210633 mutants were constructed with a similar allelic exchange system to *V. cholerae* as previously described, with the suicide vector pDM4^40^.

### Growth assays

#### Growth curves in liquid medium

For automated *A*_600_ measurements, 1 ml of a saturated 37 °C LB overnight *V. cholerae* culture was washed once with 1 ml fresh media (specific to the experiment) and resuspended in 1 ml fresh media. Resuspended bacteria were diluted to a starting dilution of 1:4,000 by performing a 1:100 dilution into 1 ml fresh media and a subsequent 1:40 dilution into 195 µl of specific media aliquoted into sterile 96-well plates (Corning). At least three technical replicates per strain and media condition were run per plate along with at least three blank media wells. Growth curves were performed in a BioTek Epoch2 spectrophotometer with shaking and *A*_600_ readings were taken every 10 min for 20–24 h. Gen5 version 3.08 (BioTek) was used for data collection. Data for each condition were averaged across technical and biological replicates corrected against the baseline blank *A*_600_ values.

#### Overexpression vector plate dilution assays on solid medium

*V. cholerae* strains were grown overnight at 37 °C in LB with addition of 50 µg ml^−1^ carbenicillin for strains with pBAD18. The cultures were diluted 1:100 into 5 ml of LB (supplemented with 50 µg ml^−1^ carbenicillin and 0.2% arabinose for strains with pBAD18) and grown at 37 °C to an OD_600_ of ~0.7. Cells were normalized to an *A*_600_ of 0.1 and then serially tenfold diluted 6 times. Five microlitres of the dilution series were plated on LB agar 100 mM Tris pH 9 (with 0.2% arabinose, 0.2% glucose, or no sugar added) and incubated at 30 °C for 18–24 h.

*S. aureus* strains were grown overnight at 37 °C in TSB with addition of 10 µg ml^−1^ erythromycin for strains with pLOW. The cultures were diluted 1:100 into 5 ml of TSB (supplemented with 10 µg ml^−1^ erythromycin and 1 mM IPTG for strains with pLOW) and grown at 37 °C to an *A*_600_ of ~0.7. Cells were normalized to an *A*_600_ of 0.1 and then serially tenfold diluted 6 times. Five microlitres of the dilution series were plated on TSA 100 mM Tris pH 9 (with or without 1 mM IPTG) and incubated at 37 °C for 18–24 h.

### Live and single timepoint phase-contrast microscopy

For single timepoint imaging of live cells, samples from the indicated cultures were concentrated as necessary and immobilized on 0.8% agarose pads in sterile PBS on glass slides (Gene Frames, Thermo) and dried before coverslip placement. For time-lapse imaging of live cells, samples were spotted on 0.8% agarose pads in sterile LB before imaging in a temperature-controlled chamber at a rate of 1 frame per minute for 3 h of growth. Cells were imaged with a Nikon Eclipse Ti microscope equipped with an Andor NeoZyla camera and a 100× oil phase 3 1.4-numerical-aperture (NA) objective using NIS Elements AR software version 4.6 (Nikon). Image analysis was performed with ImageJ version 1.53. Images in manuscript figures are representative of at least 10 fields of view (FOVs) from the same sample (>200 cells) and multiple independent replicate cultures as indicated.

### Sphere formation and incubation assays

To induce sphere formation, *V. cholerae* 37 °C overnight cultures (where Δ*vca0040* cells are still largely rod-shaped) were back-diluted 1:100 into the indicated supernatant or fresh media and grown for 4 h shaking at 200 rpm at 30 °C. Then, cells were imaged as described above. For the d/l-Ala treatment assay, overnight cultures were expanded in fresh LB for 90 min prior to spike-in of the amino acid for 1 h.

### MIC assays

#### *V. cholerae*

To quantify MICs for various antimicrobial agents, 37 °C overnight cultures of *V. cholerae* were diluted 1:100,000 in fresh media. Diluted cultures were used to inoculate 96-well plates containing 12 twofold dilutions of the indicated agent in LB medium at a ratio of 50 µl culture:50 µl medium. Four technical replicates were performed per strain per dilution. Plates were incubated for 24 h at 37 °C and MIC values were read as the first dilution where no turbidity was observed. For repeat assays, MICs were performed with independent overnight cultures.

#### *S. aureus* and *E. coli*

Overnight cultures (at 37 °C) of *S. aureus* or *E. coli* were diluted to *A*_600_ = 0.01 and then further diluted 1:100 in fresh TSB media. Diluted cultures were added to 96-well plates containing twofold dilutions of the indicated agent in TSB medium or TSB medium 5 mM Tris pH 8.5 at a ratio of 75 µl culture:75 µl media. Plates were incubated for 24 h at 30 °C (37 °C for *E. coli*) with shaking. MIC values were read off as the first dilution where no turbidity was observed.

For *E. coli lptD4213* vector overexpression MICs, overnight cultures were diluted 1:100 into non-buffered LB + carbenicillin + 0.2% arabinose. The culture was outgrown for 1.5–2 h and diluted to *A*_600_ = 0.01. This was further diluted to *A*_600_ = 0.0001 and 75 µl of the culture was added to 75 µl of serial dilution of the drug. Diluted cultures were added to 96-well plates containing 2-fold dilutions of the indicated agent in is LB 5 mM Tris pH 8.5, 1 mM CaCl_2_, with 0.2% glucose or 0.2% arabinose at a ratio of 75 µl culture: 75 µl media. Plates were incubated and read as described above.

### Peptidoglycan characterization

#### Preparation of *V. cholerae* and *S. aureus* sacculi

For *V. cholerae*, strains were grown overnight at 37 °C in LB + 200 µg ml^−1^ streptomycin. For each sample, 500 µl of overnight culture were collected and centrifuged at 5,000*g* for 5 min, washed once with 500 µl of the corresponding media, and resuspended in 500 µl of the corresponding media. This culture was then added to 50 ml of M63 media (pH 7 with 100 mM NaCl, pH 8 with 100 mM NaCl or pH 8 with 0 mM NaCl) supplemented with 2% glucose and 200 µg ml^−1^ streptomycin and incubated at 30 °C for approximately 6 h until the *A*_600_ reached ~0.5. Bacteria were collected by centrifugation at 6,000*g* for 10 min at 4 °C and resuspended in 1.5 ml of 1× PBS. The resuspension was added dropwise into 1.5 ml of boiling 5% SDS solution. The mixture was boiled for 1 h and stirred for a further 2 h after the heat was turned off.

For *S. aureus*, strains were grown overnight at 37 °C in 3 ml TSB. 500 µl of the overnight culture was added to 100 ml of TSB without pH adjustment or TSB + 100 mM bicine pH 8.5 and incubated at 37 °C for ~2 h until the *A*_600_ reached 0.5. The bacterial cells were collected by centrifugation at 5,000*g* for 10 min at 4 °C and resuspended in 1.5 ml of 1× PBS. Resuspended samples were boiled as for *V. cholerae*.

#### Total peptidoglycan and cross-linking quantification from sacculi samples

Peptidoglycan was extracted from boiled samples as described previously for Gram-negative and Gram-positive bacteria^55^. Once boiled, cell wall material was pelleted by ultracentrifugation and washed with water. Clean sacculi were digested with muramidase (100 μg ml^−1^) and soluble muropeptides reduced using 0.5 M sodium borate pH 9.5 and 10 mg ml^−1^ sodium borohydride. The pH of the samples was then adjusted to 3.5 with phosphoric acid. UPLC analyses were performed on a Waters UPLC system equipped with an ACQUITY UPLC BEH C18 Column, 130 Å, 1.7 μm, 2.1 mm × 150 mm (Waters Corporation) and identified by *A*_204 nm_. Muropeptides were separated using a linear gradient from buffer A (0.1% formic acid in water) to buffer B (0.1% formic acid in acetonitrile). Identification of individual peaks was assigned by comparison of the retention times and profiles to validated chromatograms. The relative amount of each muropeptide was calculated by dividing the peak area of a muropeptide by the total area of the chromatogram. The abundance of peptidoglycan (total peptidoglycan) was assessed by normalizing the total area of the chromatogram to the *A*_600_. The degree of cross-linking was calculated as described previously^56^.

#### Intracellular UDP-M5 quantification

For *V. cholerae*, strains were grown overnight at 37 °C in LB + 200 µg ml^−1^ streptomycin. For each sample, 500 µl of overnight culture were collected and centrifuged at 5,000*g* for 5 min, washed once with 500 µl of the corresponding media, and resuspended in 500 µl of the corresponding media. Eighty microlitres of this resuspension was then added to 8 ml of M63 media (pH 7 with 100 mM NaCl, pH 8 with 100 mM NaCl or pH 8 with 0 mM NaCl) supplemented with 2% glucose and 200 µg ml^−1^ streptomycin and incubated at 30 °C for approximately 6 h until the *A*_600_ reached ~0.5. Bacteria cells were collected by centrifugation at 5,000*g* for 10 min at 4 °C, washed twice with 1 ml of ice cold 0.9% NaCl, and resuspended in 200 µl of milliQ water. The resuspension was boiled for 30 min, centrifuged at 20,000*g* for 15 min, and the supernatant was filtered and analysed.

For *S. aureus*, strains were grown overnight at 37 °C in 3 ml TSB. Eighty microlitres of the overnight culture was added to 8 ml of TSB without pH adjustment or TSB + 100 mM bicine pH 8.5 and incubated at 37 °C for ~2 h until the *A*_600_ reached ~0.5. Cultures were then processed as for *V. cholerae*.

Quantification of soluble UDP-M5 muropeptide levels by liquid chromatography–mass spectrometry (LC–MS) of filtered supernatants was performed as previously described^57^. Detection and characterization of soluble muropeptides by LC–MS was performed on an UPLC system interfaced with a Xevo G2/XS Q-TOF mass spectrometer (Waters Corporation) using previously reported conditions^57^. UDP-M5 levels were quantified by integrating peak areas from extracted ion chromatograms (EICs) of the corresponding *m*/*z* value. UDP-M5 abundance was normalized to culture *A*_600_ as for total peptidoglycan.

### Quantification of C55-OH and C55-P in bacterial membrane lipid extracts

#### Preparation of *S. aureus* or *V. cholerae* membranes

*S. aureus* or *V. cholerae* cells were grown overnight at 37 °C in 10 ml TSB or LB pH 7, respectively. Cells were washed, collected by centrifugation, resuspended in the experimental medium (for example, TSB or LB at a given pH) and inoculated at a 1:200 dilution into 200 ml of the experimental medium. Once an *A*_600_ of 0.6 was reached, cells were collected by centrifugation, resuspended in 10 ml of phosphate-buffered saline (PBS), and disrupted in a French press. Cell debris was discarded, and the supernatant centrifuged in a Beckman Optima Max TL ultracentrifuge for 15 min at 270,000*g*. Lipids from the membrane pellets were then extracted as described below.

#### Extraction of lipids from membrane fractions

*S. aureus* and *V. cholerae* membrane lipids were extracted largely as previously described^58^. Membrane pellets were resuspended in 500 µl of PBS. Then, 1.25 ml of methanol and 625 µl of chloroform were added. The suspension was vortexed for 2 min at room temperature, and the homogenates were centrifuged at 7,100*g* for 10 min at 4 °C. 625 µl of chloroform and 625 µl of PBS were added to the supernatants and they were vortexed and centrifuged at 7,100*g* for 10 min at 4 °C to separate the chloroform phase from the PBS–methanol phase. The chloroform phase was then isolated and vacuum dried. Dried pellets containing the purified membrane lipids were resuspended in 300 µl of the UPLC mobile phase solvents (50% H_2_O with 0.1 % formic acid + 50% isopropanol with 0.1 % formic acid). To ensure that this method is capable of extracting C55-OH and C55-P, 200 nmol of each lipid standard (Larodan) was added to fresh purified membrane samples before subjecting them to the extraction protocol.

#### Detection of C55-OH and C55-P by UPLC

Lipids extracts resuspended in UPLC mobile phase solvents were analysed by UPLC on a reverse-phase C18 column Kinetex C18 UPLC Column 1.7 µm particle size, 100 Å pore size, 50 × 2.1 mm. Separation of the lipids was accomplished using a gradient of H_2_O with 0.1% formic acid for solvent A and isopropanol with 0.1 % formic acid for solvent B, a flow rate of 0.4 ml/min with a linear gradient over 8 min, column temperature 60 °C and a wavelength of 210 nm. Identification of C55-OH and C55-P was based on the retention time of standards and their abundances were calculated relative to the total peak area of each chromatogram. For calculating mutant-to-wild type ratios, samples from independent cultures grown on the same day were randomly paired. Empower3 chromatography data software (Waters) was used for UPLC data collection and analysis.

### Ampho-FL synthesis and staining

To synthesize ampho-FL, 5 mg of amphomycin (Cayman Chemical) was dissolved in 200 µl of dimethyl formamide (DMF) and combined with 7 µl of triethylamine. Separately, 3 mg of fluorescein-C_5,6_-NHS (Thermo Fisher, USA) was dissolved in 200 µl of DMF and then added to the amphomycin solution. The reaction mixture was stirred at room temperature in the dark for 24 h. The solution was diluted in one equal volume of DMSO and purified by reverse-phase HPLC (Agilent 1260 Infinity) using a C18 stationary phase column (Luna 5 µM C18(2) 100 Å, 250 × 10 mm). HPLC conditions were as follows—phase A: water (0.1% formic acid); phase B: acetonitrile (0.1% formic acid). Phase B: 0–2 min, 50%; 2–15 min, linear gradient 50%–100%, 15–17 min, 100%. The wavelength of the detector was set at 254 nm. The flow rate was 4.7 ml min^−1^. The mixture of conjugated fluorescein-C_5,6_ products eluted at 9 min. HPLC eluates were collected in a 50 ml round-bottom flask, concentrated by rotatory evaporation, transferred with DMSO to a tared microcentrifuge tube and lyophilized, resulting in 1.7 mg of ampho-FL (M+1 = 1,649.23).

For ampho-FL labelling, overnight cultures of *S. aureus* HG003 wild type or ∆*SAOUHSC_0084*6 were diluted 1:100 in fresh TSB media buffered at pH 6 (100 mM MES), 7 (100 mM Tris), or 8.5 (100 mM bicine). The new cultures were grown to *A*_600_ = 0.5. 1 ml of the culture was centrifuged at 1,000*g* for 5 min. The pellet was resuspended in 500 µl of 1× Tris-buffered saline (TBS) pH 9. Ninety-eight microlitres of the mixture was transferred to a new tube and combined with 2 µl of 10 mg ml^−1^ ampho-FL conjugate in DMSO. The mixture was incubated in the dark at room temperature for 10 min, washed three times with 500 µl of 1× TBS pH 9, and resuspended in 100 µl of 1× TBS pH 9. Ten microlitres bacteria was then added to a 0.8% agarose pad in TBS pH 9 containing 1 μg ml^−1^ propidium iodide and imaged as described above. To avoid bias in FOV selection, the pad was scanned with the FITC channel off to select FOVs based solely on cell density. Bacterial cells were imaged using DIC at a 30 ms exposure and ampho-FL signal was captured with the FITC filter at a 1 s exposure. Fluorescence intensity of ampho-FL was quantified using ImageJ version 1.53. FITC signal profiles were generated by drawing bisecting lines through dividing, propidium iodide-negative cells. The FITC signal was recorded at the background to the left of the cells (*A*), at the left wall peak (*B*), at the middle walls (*C*), at the right wall peak (*D*), and at the background to the right of the cells (*E*). The average signal was calculated by (*B* + *C* + *D*)/4 − (*A* + *E*)/2.

### Transposon-insertion sequencing

Generation of transposon libraries in the Δ*vca0040* and Δ*yghB* backgrounds was performed as previously described for HaitiWT *V. cholerae*^15^. Strains were conjugated to SM10λpir *E. coli* bearing the donor transposon vector pSC189. Due to the moderate conjugation defect of Δ*vca0040*, we used a 5× concentration of the conjugation reactions compared to the other libraries. Reactions were plated on 245 mm^2^ LB + streptomycin/kanamycin agar plates to isolate *V. cholerae* transconjugants and incubated overnight at 30 °C. Two independent Δ*vca0040* and Δ*yghB* libraries were generated, consisting of approximately 200,000 colonies each and stocked at an *A*_600_ of ~10 in LB + 25% glycerol. For synthetic transposon-insertion sequencing analyses, a frozen aliquot of each library was thawed and used for genomic DNA extraction with the Wizard kit (Promega). DNA libraries were prepared in an identical manner to previous transposon-insertion sequencing experiments from our group and sequenced on an in-house MiSeq platform (lllumina)^18^. Reads were trimmed, mapped, and processed as previously described with the Con-ARTIST transposon-insertion sequencing analysis pipeline using Python version 3.0 and Matlab version 9^18^. To identify synthetic interaction loci, we used the wild type as the ‘input’ library and the mutant as the ‘output’ library during analysis.

### RNA sequencing

For transcriptomic analyses, triplicate wild-type and Δ*vca0040* *V. cholerae* 37 °C overnight cultures were back-diluted 1:100 into fresh LB medium and grown at 30 °C for 8 h with shaking. At 8 h, samples were checked by microscopy to ensure onset of sphere formation in the mutant samples, at which point 2 ml of each culture was spun down (5 min at 5000*g* at room temperature). RNA was extracted with Trizol reagent (Sigma-Aldrich) per the manufacturer’s instructions. Isolated RNA was then DNase-treated and re-isolated with ethanol precipitation according to a standard protocol. RNA samples were quality checked with a Bioanalyzer to confirm RIN values > 6. Library preparation and sequencing was performed by the Microbial Genome Sequencing Center (Pittsburgh). RNA-sequencing analysis was performed largely as described^59^. Reads were mapped to the *V. cho\lerae* KW3 genome (NCBI assembly GCA_001318185.1) with Bowtie2 (version 2.4.1)^60^. A read matrix for each sample was then generated with featureCounts from Rsubread (version 2.4.3)^61^. Read matrices were then analysed with DESeq2 (version 1.30.1) using default settings in R (version 4.0.3) to identify differentially expressed genes. Data shrinkage was performed with ashr^62^.

### Multiplexed tandem mass tag proteomics

For proteomic analyses, triplicate 37 °C wild-type and Δ*secDF1* *V. cholerae* overnight cultures were grown as described in ‘RNA sequencing’. At 8 h of growth, whole-cell pellet (WCP) samples were prepared by centrifuging 1 ml of cells for 5 min at 5,000*g* at room temperature, washing once in fresh LB, flash frozen and kept at −80 °C. Mass spectrometry analysis was performed by the Thermo Fisher Center for Multiplexed Proteomics (TCMP) at Harvard Medical School according to standard protocols. WCP samples were subjected to a total proteomics workflow with fractionation. Pelleted cells were first lysed in 8 M urea, 200 mM 4-(2-hydroxyethyl)-1-piperazinepropanesulfonic acid (EPPS) and 1% SDS with phosphatase and protease inhibitors. Then, samples were reduced with DTT and alkylated with iodoacetamide. Alkylated proteins were precipitated with methanol/chloroform and resuspended in 200 mM EPPS pH 8 and digested sequentially with 1:50 LysC and 1:100 trypsin. Peptides were labelled with TMT16 reagents, pooled, and fractionated by a basic reverse-phase protocol into 12 fractions. Fractions were then dried, cleaned on a C18-packed stage tip, and eluted into an mass spectrometry sample vial for analysis. Samples were resuspended in 5% acetonitrile/5% formic acid and analysed by LC–MS3 on an Orbitrap Lumos mass spectrometer using a 180 min MS3 method. Peptides were detected (MS1) and quantified (MS3) in the Orbitrap, and sequenced (MS2) in the ion trap. MS2 spectra were searched with the COMET algorithm against the *V. cholerae* KW3 proteome, its reversed complement, and known contaminants. Spectral matches were filtered to a 1% false-discovery rate using the target-decoy strategy combined with linear discriminant analysis. Proteins were quantified from peptides with a summed signal to noise threshold of >150 and isolation specificity of >0.5.

### Spontaneous suppressor isolation and sequencing

#### *V. cholerae*

To ensure independent isolation of spontaneous suppressors, the Δ*vca0040* strain was re-derived 11 times from different conjugation reactions. From each colony PCR- and cell shape defect-verified Δ*vca0040* clone, colonies with mutant morphology (small and completely blue) on LB + streptomycin/X-gal plates were re-streaked onto new LB + streptomycin/X-gal plates and grown for 36–48 h at 37 °C. This process was repeated once. From the tertiary plates, a single colony with wild-type morphology (large with a white halo) was re-streaked to confirm a stable suppressor phenotype. Suppressors were checked by microscopy of 30 °C overnight cultures to confirm cell shape defect reversion and verified by colony PCR to confirm the absence of *vca0040* from their genome. Validated suppressors were grown overnight, and genomic DNA was extracted as described above. Library preparation and sequencing were either performed in-house or outsourced to the Microbial Genome Sequencing Center (Pittsburgh). For in-house whole-genome sequencing, gDNA was tagmented and barcoded with the Nextera XT library preparation kit (Illumina), quality checked by BioAnalyzer and sequenced on a MiSeq platform to at least 20–50× depth of the *V. cholerae* genome (~4 Mbp). Genome assembly and variant identification was performed with CLC Genomics Workbench 12 (Qiagen, Germany). Variants were filtered against an assembled HaitiWT isolate re-sequenced with the same workflow. Mutations were assigned as suppressors if they were present in >90% of reads and did not occur in a known poorly mapping or highly repetitive region such as a tRNA locus. For the secondary screen in *secDF2-* Δ*vca0040* bacteria, the Δ*vca0040* deletion was re-derived three independent times in the Δ*secD2* or Δ*secF2* background. Suppressors were isolated and sequenced identically to those in the parental Δ*vca0040* background.

#### *S. aureus*

Multiple 2 ml cultures of *S. aureus* HG003 ∆*SAOUHSC_00846* were grown in TSB overnight at 37 °C. One-hundred and ninety microlitres from each independent overnight culture was then separately plated on TSA 100 mM Tris pH 9 plates (where the mutant is entirely inhibited for growth) and grown at 37 °C for 24 h. Suppressors were confirmed by re-streaking on TSA 100 mM Tris pH 9 plates. Validated suppressors were grown overnight, and genomic DNA was extracted as described above. Library preparation, sequencing, and variant identification were performed as described above for *V. cholerae*.

### Infant rabbit *V. cholerae* infections and caecal fluid imaging

Infant rabbit oral infections with *V. cholerae* were performed as described previously^15^. One- to two-day-old New Zealand White rabbits (Charles River Laboratories) were housed with their dams for the duration of the experiment in a temperature- and humidity-controlled facility with 12-h light/dark cycles (16–22 °C, 50% humidity). Inocula were prepared by centrifuging a late exponential phase culture (*A*_600_ 0.6–0.8) *V. cholerae* and resuspending in 2.5% sodium bicarbonate. For varying the pH of the inocula, sodium bicarbonate was pH-adjusted to pH 9 or 7 with NaOH or HCl immediately before resuspension. Infected kits were were orally gavaged with 10^9^ CFU *V. cholerae* in a 500 μl gavage volume, returned to their dam, and monitored for 16–20 h post-infection, when they were sacrificed by isoflurane inhalation and intracardiac injection of 20 mEq potassium chloride. Small intestinal segments and the caecum were isolated by dissection, homogenized by bead beading in PBS, and dilutions were plated on appropriate agar plates for colony enumeration. Plates were counted after an overnight incubation at 30 °C. LOD measurements were calculated by imputing a single colony at the lowest (lower bound) or highest (higher bound) dilution where a colony could reasonably have been identified, meaning that LOD values are samples where the true value is either at most (for lower bound LODs) or at least (for higher bound LODs) the limit value. During dissection of the caecum, a 28G needle was used to extract crude caecal fluid. A sample of crude caecal fluid was taken for CFU plating and imaging, and the remainder was centrifuged at 21,000*g* for 2 min. The pellet was discarded and supernatants were frozen at −20 °C for downstream analyses. For caecal fluid incubation assays, overnight cultures of *V. cholerae* were back-diluted 1:100 in caecal fluid and grown for 4 h at 30 °C shaking before imaging.

### Mouse *S. aureus* intravenous infection

*S. aureus* HG003 (wild type) and KanR-marked Δ*SAOUHSC_00846* were grown overnight at 37 °C. Cultures were mixed at a 1:1 ratio, combined 1:1 with 50% glycerol, and stored at frozen at −80 °C in several aliquots. For infections, an aliquot was thawed and diluted in PBS to a density of ~3 × 10^7^ CFU ml^−1^. One-hundred microlitres was injected into the tail vein of 8- to 9-week-old female Swiss Webster mice. Mice were monitored daily and housed in a temperature- and humidity-controlled facility with 12-h light/dark cycles (20–24 °C, 50% humidity). At days 2 and 5 post-infection, mice were sacrificed by isoflurane inhalation and cervical dislocation and the heart, lungs, right kidney, liver and spleen were collected. Organs were homogenized with stainless steel beads in PBS and plated on TSA and TSA + kanamycin (TSAK) and incubated overnight to enumerate total Δ*SAOUHSC_00846* CFU, respectively. LOD calculations were performed as for infant rabbit experiments.

### Animal use statement

All animal work in this study was performed in accordance with the NIH Guide on Use of and Care for Laboratory Animals and with the approval of the Brigham and Women’s Hospital IACUC (protocol no. 2016N000334 for infant rabbits and protocol no. 2016N000416 for mice).

### Statistics and reproducibility

Statistical tests used and replicate information are indicated in the figure legends, relevant methods sections and the Reporting summary. Unless otherwise indicated, experiments were performed with at least three independent biological replicates and either representative results or aggregate data (mean ± s.d.) are shown. Sample sizes were not statistically predetermined. Investigators were not blinded to sample identity throughout the study, and randomization of animals in infection experiments was performed as described in the Reporting Summary. All replicates resulted in similar results. Statistical analyses were performed in Prism 9 (Graphpad) and Microsoft Excel 2020.

### Reporting summary

Further information on research design is available in the Nature Portfolio Reporting Summary linked to this article.

## Online content

Any methods, additional references, Nature Portfolio reporting summaries, source data, extended data, supplementary information, acknowledgements, peer review information; details of author contributions and competing interests; and statements of data and code availability are available at 10.1038/s41586-022-05569-1.

## Supplementary information

Reporting Summary

Peer Review File

Supplementary Table 1 Bacterial and archaeal clades with PF04018-containing proteins. Annotree was used to plot and identify species with an annotated DUF368 domain. Each tab of the spreadsheet is a different level of bacterial classification (Phylum>Order>Family>Genus>Species). Archaeal clades are combined on the tab “Archaea”.

Supplementary Table 2 Homologues of VCA0040 in bacteria. HMMER was used to identify homologous sequences to VCA0040 in the Uniprot database. Each species is listed once.

Supplementary Table 3 Notable domain architectures of DUF368- and DedA-containing proteins. Sequences were obtained by searching InterProScan with the PF family designations for DUF368 (PF04018) and DedA (PF09335).

Supplementary Table 4 RNAseq of Δ*vca0040 V. cholerae* during sphere formation. For the top 40 up-regulated genes in Δ*vca0040*, manual curation of subcellular localization was performed with SignalP and pSortb. P-values were obtained through the Wald test performed by DESeq2 with adjustments for multiple comparisons.

Supplementary Table 5 MIC data for *V. cholerae, S. aureus* and *E. coli.* Data for each organism is separated into individual worksheets. Note that *S. aureus* data is split into two worksheets, one for WT versus Δ*0846* characterization (“SA”) and one for WT versus Δ*0846*, Δ*2816* and Δ*0901* (“SA_dedA”). All *V. cholerae* data is under “VC” and all *E. coli lptd4213* data is under “EC_lptd4213”.

Supplementary Table 6 Synthetic transposon-insertion screening in Δ*vca0040 V. cholerae.* Note that intergenic regions (IG_) and regions with <5 informative sites (possible transposon insertion sites) were not analyzed. Hits on the second tab were thresholded at an inverse MWU p-value >100 before sorting by mean log_2_ fold change.

Supplementary Table 7 Synthetic transposon-insertion screening in Δ*yghB V. cholerae.* Note that this dataset has not been filtered as for Supplementary Table 6.

Supplementary Table 8 Multiplexed comparative proteomics of whole-cell WT and Δ*secDF1 V. cholerae*. Fold change was calculated by dividing the average normalized relative abundance of proteins in Δ*secDF1* by that of WT.

Supplementary Table 9 Conservation of PF04018, PF09335, and PF02763 in bacterial species. Spreadsheet was generated by Annotree queries for all possible combinations of the three protein families. The “Master” sheet lists all species-level organisms with the indicated combination of domains. The “Model Species” sheet lists selected microorganisms of particular interest and can be used to look up any given species in the reference table. Species of interest should be manually confirmed for presence or absence by BLAST to guard against misannotation or non-annotation issues.

Supplementary Table 10 Strains and vectors and genomic information used in this study. Any listed strains or vectors may be requested from the lead contact (mwaldor@research.bwh.harvard.edu).

Supplementary Table 11 BLAST dictionary for assignment of putative VC names to HaitiWT *V. cholerae* loci. All coding sequences from HaitiWT were used as queries in a batch BLAST of the N16961 *V. cholerae* proteome. The top hit by e-value was selected and VC name automatically assigned. However, as batch BLAST outputs a hit regardless of confidence, manual curation is still required for specific loci when using this dictionary.

## Data Availability

Sequence data were deposited to the NCBI Sequence Read Archive under BioProject accession codes PRJNA868324 (*V. cholerae* suppressor genome sequencing), PRJNA868332 (*V. cholerae* RNA sequencing), PRJNA877769 (*V. cholerae* transposon-insertion sequencing) and PRJNA877773 (*S. aureus* suppressor genome sequencing). Proteomics data were deposited in MassIVE under the accession code MSV000090217. All other data are freely available without restriction from the corresponding authors upon request. Source data are provided with this paper.

## Source data

Source Data Fig. 5
Source Data Extended Data Fig. 8
Source Data Extended Data Fig. 9
