# Supplementary material for: Undecaprenyl phosphate translocases confer conditional microbial fitness
Source: Nature. 2022 Nov 30;613(7945):721–8. doi: 10.1038/s41586-022-05569-1 (PMC9876793; doi:10.1038/s41586-022-05569-1)
Supplement: Supplementary file 1 — Reporting Summary [file 41586_2022_5569_MOESM1_ESM.pdf]

nature portfolio

Corresponding author(s): Matthew K. Waldor

Last updated by author(s): 11/8/2022

Reporting Summary

Nature Portfolio wishes to improve the reproducibility of the work that we publish. This form provides structure for consistency and transparency in reporting. For further information on Nature Portfolio policies, see our Editorial Policies and the Editorial Policy Checklist.

Statistics

For all statistical analyses, confirm that the following items are present in the figure legend, table legend, main text, or Methods section.

n/a	Confirmed	
☐	☒	The exact sample size (*n*) for each experimental group/condition, given as a discrete number and unit of measurement
☒	☐	A statement on whether measurements were taken from distinct samples or whether the same sample was measured repeatedly
☐	☒	The statistical test(s) used AND whether they are one- or two-sided<br>*Only common tests should be described solely by name; describe more complex techniques in the Methods section.*
☒	☐	A description of all covariates tested
☒	☐	A description of any assumptions or corrections, such as tests of normality and adjustment for multiple comparisons
☒	☐	A full description of the statistical parameters including central tendency (e.g. means) or other basic estimates (e.g. regression coefficient) AND variation (e.g. standard deviation) or associated estimates of uncertainty (e.g. confidence intervals)
☐	☒	For null hypothesis testing, the test statistic (e.g. *F*, *t*, *r*) with confidence intervals, effect sizes, degrees of freedom and *P* value noted<br>*Give P values as exact values whenever suitable.*
☒	☐	For Bayesian analysis, information on the choice of priors and Markov chain Monte Carlo settings
☒	☐	For hierarchical and complex designs, identification of the appropriate level for tests and full reporting of outcomes
☒	☐	Estimates of effect sizes (e.g. Cohen's *d*, Pearson's *r*), indicating how they were calculated

Our web collection on statistics for biologists contains articles on many of the points above.

Software and code

Policy information about availability of computer code

Data collection	Gen5 version 3.08 (Biotek) was used to collect OD600 measurements for growth curves. Empower3 Chromatography Data Software (Waters) was used to collect and analyze UPLC measurements. NIS-Elements AR version 4.6 (Nikon) was used for collection of microscopy data. High-throughput sequencing data were collected with MiSeq on-board Illumina Experiment Manager software.
Data analysis	Graphpad Prism 8.0 and Microsoft Excel 2020 were used for graphing and analyzing most data. CLC Genomics Workbench 12 (Qiagen) was used for analyzing bacterial genome sequencing data. Microscopy images were analyzed with ImageJ version 1.53. RNAseq data were analyzed with R version 4.0.3, Bowtie2 version 2.4.1, Rsubread version 2.4.3, and DESeq2 version 1.30.1. Python version 3.0 and Matlab version 9 were used to analyze transposon-insertion sequencing data.

For manuscripts utilizing custom algorithms or software that are central to the research but not yet described in published literature, software must be made available to editors and reviewers. We strongly encourage code deposition in a community repository (e.g. GitHub). See the Nature Portfolio guidelines for submitting code & software for further information.

Data

Policy information about availability of data

All manuscripts must include a data availability statement. This statement should provide the following information, where applicable:

- Accession codes, unique identifiers, or web links for publicly available datasets
- A description of any restrictions on data availability
- For clinical datasets or third party data, please ensure that the statement adheres to our policy

Sequencing data were deposited to the NCBI SRA under the following BioProject accession codes: PRJNA868324 (V. cholerae suppressor genome sequencing), PRJNA868332 (V. cholerae RNAseq), PRJNA877769 (V. cholerae transposon-insertion sequencing), and PRJNA877773 (S. aureus suppressor genome sequencing).

Proteomics data were deposited in MassIVE under the accession code MSV000090217. Source data for animal experiments is provided in an attached spreadsheet. All other data is freely available without restriction from the corresponding authors upon request.

Field-specific reporting

Please select the one below that is the best fit for your research. If you are not sure, read the appropriate sections before making your selection.

☒ Life sciences ☐ Behavioural & social sciences ☐ Ecological, evolutionary & environmental sciences

For a reference copy of the document with all sections, see nature.com/documents/nr-reporting-summary-flat.pdf

Life sciences study design

All studies must disclose on these points even when the disclosure is negative.

Sample size	No sample size calculations were performed; sample sizes were chosen based on prior in vitro (e.g., PMID: 31289173) and in vivo (e.g., PMID: 29899024) studies using V. cholerae.
Data exclusions	No data were excluded from analysis.
Replication	All individual points in graphs are independent biological replicates (i.e., individual animals or independent overnight cultures used to inoculate experimental cultures). Images of plates are representative of at least three independent replications.<br><br>Experiments were replicated as follows:<br>Figure 1: Three independent overnight cultures were used in each panel apart from A-D. Growth curve data in Panels E and H shows a representative experiment of three biological replicates.<br>Figure 2: Three independent overnight cultures were used in each panel. Panel C shows a representative result of three experiments.<br>Figure 3: Panel A shows at least three independent MIC cultures for each antibiotic. All traces shown are representative of three independent biological replicates. The replicates for Panel C and F are quantified in panel D/E and G/H respectively. The image in Panel I is representative of at least three independent cultures used for ampho-FL imaging, which are quantified in Panel J<br>Figure 4: Panel A is a single transposon-insertion sequencing experiment combining two independently generated libraries. Panel C and E are representative of three independent biological replicates. Three independent biological replicates are shown in Panel F.<br>Figure 5: All points graphed in this figure represent individual rabbits. Points in Panel E derive from rabbits in Panel D. Panel F depicts all rabbits used. Columns in Panel G are individual biological replicates.<br>ED Figure 1: Panel A is from a previously published dataset from a single rabbit.<br>ED Figure 2: N/A<br>ED Figure 3: Panel A shows means from three independent cultures. All other panels with the exception of Panel D show representative results from at least three independent overnight cultures. Panel D is a representative timelapse of >20 spherical cells from three different fields of view from a single overnight culture.<br>ED Figure 4: Panels A and B are representative results from three independent biological replicates. Panels C-G depict individual biological replicates (each as a point).<br>ED Figure 5: Panel B shows representative images from three independent cultures used for RNAseq, which are plotted in Panel C and aggregated in Panel D. Panel E shows representative MICs, with replicate values listed in Supplementary Table 5 of 3 independent experiments. Panel F shows representative traces from three independent biological replicates that are quantified in Panel G. Panel H shows representative images that are quantified in Panel I. For Panel I, three independent cultures were imaged at pH 6 and 7 and a single culture was imaged at pH 8.5.<br>ED Figure 6: Panel A is a single transposon-insertion sequencing experiment combining two independently generated libraries. Panels B and C are representative of three independent biological replicates.<br>ED Figure 7: Panel A shows representative images of suppressor colony isolation of one of 16 independently isolated lines. Panel C, F, L and M shows representative results of three independent biological replicates. Panel H shows aggregated proteomics data comparing four independent WT replicates against three independent mutant replicates. Panels G-K show growth curves representative of three independent biological replicates. pH 8 0 and 100mM curves are plotted in both Panel I and Panels J/K for clarity. Panels L and M are representative of three independent biological replicates.<br>ED Figure 8: Panels A and B show individual rabbits (same as Main Figure 4B). Panel C and D show representative images from singly-infected rabbits, where each row is a selected rabbit.<br>ED Figure 9: All points graphed are individual mice. Panel B and C show different analyses of the same CFU data (n=5 mice in each group).<br>ED Figure 10: Panel B is representative of three independent biological replicates.<br><br>All attempts at replication were successful.
Randomization	For in vivo studies, infant rabbits within a litter were allocated to different infection groups to achieve an equivalent mean animal weight in each group. Prior studies in this system have demonstrated this randomization is sufficient to control for other covariates (e.g., PMID: 20689747 and 29899024). For mouse infections, mice were randomly allocated to receive a given inoculum. For in vitro studies, no specific randomization processes were neccessary as experiments were performed with overnight cultures from which experimental samples were taken.
Blinding	Blinding was not performed as all measurements in the study were quantitative at defined timepoints, meaning that additional blinding would not have affected the results. For microscopy FOV selection, blinding was not necessary because the phenotypes were either uniform, or FOV searching was based on cell density and not fluorescence signal (i.e., for ampho-FL).

Reporting for specific materials, systems and methods

We require information from authors about some types of materials, experimental systems and methods used in many studies. Here, indicate whether each material, system or method listed is relevant to your study. If you are not sure if a list item applies to your research, read the appropriate section before selecting a response.

Materials & experimental systems

n/a	Involved in the study
☒	☐ Antibodies
☒	☐ Eukaryotic cell lines
☒	☐ Palaeontology and archaeology
☐	☒ Animals and other organisms
☒	☐ Human research participants
☒	☐ Clinical data
☒	☐ Dual use research of concern

Methods

n/a	Involved in the study
☒	☐ ChIP-seq
☒	☐ Flow cytometry
☒	☐ MRI-based neuroimaging

Animals and other organisms

Policy information about studies involving animals; ARRIVE guidelines recommended for reporting animal research

Laboratory animals	Rabbits:<br>2-3-day old infant New Zealand White rabbits of both sexes were used for V. cholerae infections. Kits were housed with their dams for the duration of the experiment in a temperature and humidity controlled facility with 12 hour light/dark cycles (61-72F, 50% humidity). Kits were sacrificed by isoflurane inhalation and intracardiac injection of 20mEq potassium chloride. Dams were anesthetized by IM injection of 150mg ketamine (Ketaset) and 50mg xylazine (Xylamed) and euthanized by IV injection of 390mg sodium pentobarbital (Euthasol).<br><br>Mice:<br>For S. aureus infections, 8-9-week-old female Swiss-Webster mice were used. Mice were housed in a temperature and humidity controlled facility with 12 hour light/dark cycles (68-75F, 50% humidity). Mice were sacrificed by isoflurane inhalation and cervical dislocation.
Wild animals	The study did not involve wild animals.
Field-collected samples	The study did not involve field-collected samples.
Ethics oversight	All animal work in this study was performed in accordance with the NIH Guide on Use of and Care for Laboratory Animals and with the approval of the Brigham and Women's Hospital IACUC (Protocol #2016N000334 for infant rabbits and #2016N000416 for mice).

Note that full information on the approval of the study protocol must also be provided in the manuscript.
