# Supplementary material for: Undecaprenyl phosphate translocases confer conditional microbial fitness
Source: Nature. 2022 Nov 30;613(7945):721–8. doi: 10.1038/s41586-022-05569-1 (PMC9876793; doi:10.1038/s41586-022-05569-1)
Supplement: Supplementary file 2 — Peer Review File [file 41586_2022_5569_MOESM2_ESM.pdf]

**Peer Review File**

**Manuscript Title:** Candidate undecaprenyl phosphate translocases enable conditional microbial fitness

## Reviewer Comments & Author Rebuttals

### Reviewer Reports on the Initial Version:

Referees' comments:

Referee #1 (Remarks to the Author):

Bacteria synthesize precursors of peptidoglycan cell wall and other envelope glycopolymers in the cytoplasm bound to the lipid carrier undecaprenyl-phosphate (C55-P). These precursors are flipped across the cytoplasmic membrane and, once the precursor moiety is used, the C55-P carrier is transported back across the membrane so that C55-P can be recycled. This recycling is an essential process (as inferred by the loss of viability caused by the antibiotic bacitracin) that occurs through an unknown mechanism. This study by Sit et al. focuses on identifying candidate C55-P transporters. The authors propose that members of the DUF368 and DedA protein families function as C55-P transporters. The authors showed that Vibrio cholerae and Staphylococcus aureus mutants lacking members of the DUF368 family exhibit growth and peptidoglycan defects in alkaline pH conditions. In Vibrio, these defects were suppressed by lowering the Na+ concentration in the media and by suppressors that presumably have reduced Na+ import into the cytoplasm. The authors also showed synthetic interactions between genes encoding both putative transporters (DUF368 and DedA). Furthermore, in Vibrio, the single dedA mutant cannot grow in NaCl-free media and the synthetic lethality between DUF368 and dedA is suppressed by acidic pH. Consequently, the authors propose that DUF368 proteins, DedA, and a yet-to-be-identified protein(s) are C55-P transporters. They also conclude that the function of these transporters is determined by environmental conditions. Specifically, Sit et al. propose that DUF368 requires sodium ions to function, while DedA and the yet-to-be-identified protein(s) require protons.

The manuscript deals with a very important question. The data are of high quality, the work is extensive, and many of the main conclusions are supported by the data with the notable exception that the authors do not show direct evidence that these proteins are C55-P transporters. The authors establish a connection between DUF368 and cell wall defects, in agreement with the proposed function. However, a direct link between these proteins and C55-P transport is missing. This is an important issue given that previous studies have reported that the loss of DedA proteins results in pleiotropic defects.

Specific major issues:
1) The study does not present evidence showing a direct link between C55-P and these putative transporters. Are C55-P levels changed in the mutants because it cannot be recycled? Can the phenotypes associated with the loss of these transporters be rescued by increasing C55-P production? Do the proteins bind C55-P? The loss of these proteins could be affecting peptidoglycan synthesis indirectly. For example, is the loss of these proteins leading to peptidoglycan defects

because their loss alters membrane potential, which can then affect peptidoglycan synthesis in other ways (such as affecting lipid II transport and/or C55-P transport by the actual transporter)? Answering some of these questions would provide stronger support of their model.

2) The authors link these proteins to C55-P indirectly by using the antibiotic amphomycin. They show that the mutant is specifically sensitive to this drug, which binds C55-P. They also see an increase in fluorescence in S. aureus mutants lacking DUF368 when using fluorescently labeled amphomycin. What is the evidence showing that amphomycin is only present on the extracytoplasmic side of the membrane? In addition, the sensitivity and fluorescence results could also be explained by proposing that the DUF368 protein directly or indirectly affects the efflux of amphomycin. Can this alternative interpretation be ruled out?

3) The authors show synthetic genetic interactions between genes encoding DUF368 and DedA proteins. Are the double mutant cells exhibiting cell wall defects? Is that why they die? The authors should show more detailed characterization of the synthetic phenotypes. At the very least, they should show growth curves and microscopy data linking peptidoglycan defects to the inability to grow.

4) Can the inability of the yghB (dedA paralog) single mutant to grow in the absence of NaCl be rescued by low pH? According to their model, suppression would be expected.

5) In V. parahaemolyticus, the DUF368 protein VPA1624 is essential even at neutral pH (Figure S10B). Does this mean that the DedA homolog YghB does not perform the same function? Or is yghB not expressed under those conditions? Or is there another protein transporting C55-P? This finding seems to contradict their model and requires further experimentation.

Minor points:

1) Figure 1H, Figure 2C, G, and H should include complementation data.

2) Please include a scale bar in Fig. 2G. Also, it would be nice to compare data for the ΔSAOUHSC_00846 S. aureus mutant at acidic and neutral pH for fluorescein-conjugated amphomycin (ampho-FL).

3) Fig. 3D and F: What are the dilutions shown in the spot assays?
4) Table S4: At what pH were the MIC assays done for the wildtype and the Δvca0040 mutant of V. cholerae? They should have been done at alkaline pH to be relevant.

5) Line 163-162: "Na+-free, but not K+-free conditions suppressed the alkaline growth and PG defects of Δvca0040 V. cholerae (Figs. 2A, B, 3A, Extended Data Figs. 5C, 6I, J)." This statement is misleading. Figs. 2A and 3A show that, but Fig. S6J shows that the mutant grows better at 5, 10, and 50 mM Na+ salt than at 0 mM Na+ (it does worse at 100 and 200 mM).

6) It might be better to test salt dependency of both transporters by using various Na+ concentrations (like in Extended Data Figure 6J) in a Δvca0040 strain complemented by either

VCA0040 or YghB.

Referee #2 (Remarks to the Author):

In their wide ranging and comprehensive work on an unknown, conserved protein in the DUF368 family, essential for V. cholera pathogenesis, Waldor and colleagues establish that it likely performs Und-P recycling, a critical step in the generation of envelope glycopolymers. Previously, no protein was associated with performing this function. Their comprehensive analysis provides rigorous in vivo evidence that this protein does perform Und-P recycling, and lacks only in vitro analysis (which is likely beyond the scope of this work) to fully establish its role. In the course of their studies, this group also showed that knocking-out a homologous gene in the gram positive organism S. aureus causes very similar phenotypes, establishing that the function spans the gram positive/negative divide. Examining DUF368 function in S. aureus also allowed them to provide further evidence of its role by showing that its phenotypes are consistent and synergistic with those of amphomycin, known to prevent recycling, and that loss of this protein confers enhanced sensitivity to amphomycin. Additionally, the group examined its regulation, and function in varying contexts including pathogenesis, indicating multifaceted regulation and redundancy, as expected from a critical cellular function. Finally, using several complementaty transposon screens, they identified and validated a synthetic lethal interaction with a DedA protein, thereby identifying a second family of proteins to carry out this function. This already strong paper would have greater impact with the addition of one experiment to generalize their findings.

1. Solidifying the S. aureus phenotypes. The authors already show that the DUF368 protein in S. aureus functions similarly to the V. cholera one, and that overexpression of 2 S. aueus DedA proteins rescued the DUF368 deletion phenotype. However, they do not report whether deletion of all DedA proteins in addition to DUF368 is possible and what the phenotypes of the triple mutant are. This is important to their framing of the broader scope of the ms,--showing that at least under some conditions, the proteins they identify have synthetic lethal phenotypes, consistent with loss of Und-P recycling upon loss of these functions.

2. It seems important to highlight bacitracin phenotypes, as they are immediately upstream of the step impacted by these new enzymes, and phenotypes are consistent with their model.

Minor

-L26 “PG biosynthesis and structure are responsive to shifting environmental conditions…” There are 4 refs here, all published in 2019-2020, all with the same first and last author. Is this really the extent of the field?

-L120. I’m not sure that this is the best citation for the MoA of amphomycin.

Referee #3 (Remarks to the Author):

This is an important paper, presenting a significant advance.

Comments:

Lines 56-58: This sentence should be re-worked to include information on why BacA is not a credible C55-P translocase candidate.

Lines 127-129: The ampho-FL labeling (Fig 2G, H) was done at alkaline pH 9 – the extent of labeling would be expected to be pH dependent based on the other phenotypes reported. This should be explicitly shown by doing the staining at acidic pH.

Lines 138-139: The docking data are of mixed value. Docking is performed under highly non-natural conditions, and demonstration of a lock and key arrangement of VCA0040 with C55-P seems to run counter to a transport function. How would such a perfectly fitted lipid be transported?

Line 179: Does the yghB mutant exhibit a growth defect at pH>8 in sodium replete conditions?

Line 191: The eukaryotic DedA proteins TMEM41B and VMP1 have been shown to scramble glycerophospholipids – there are no data to indicate lipid binding.

Line 193: Because the double mutant (vca0040, yghB) grows under acidic conditions, the authors conclude that there must be at least one other protein responsible for C55-P translocation. This is reasonable. However, protonation of the phosphate group under suitably acidic conditions would promote spontaneous scrambling of C55-P, akin to that reported for phosphatidic acid (PMID1993189). This possibility should be discussed with appropriate citation.

Related to line 193: Under alkaline conditions, the vca0040 mutant grows in low sodium but the yghB mutant does not. And vice versa. The model in Extended Data Figure 6E suggests the differential roles of the Sec mutant suppressors, with the implication that Na- or proton-motive force is used differentially to support one or other of the C55-P translocases – the effect could be direct or indirect. Much is made of the idea that environmental conditions control which translocase is relevant, but what would be the evolutionary advantage in this system? One environmentally insensitive translocase should do the trick. There is no apparent redundancy in the Lipid II translocation machinery, so why would the recycling of C55-P require redundancy? It would be worth discussing these points.

The paper describes comprehensive genetics-based analyses but lacks a direct test of the C55-P transport activity of the VCA0040 and YghB proteins. While such a test is beyond the scope of the paper, the authors should point out that a definitive conclusion about the role/activity of the

candidate translocases requires a biochemical test using purified proteins.

The TMEM41B and VMP1 DedA proteins in eukaryotes have been shown to be glycerophospholipid scramblases. The implication in this paper is that the candidate C55-P transporters couple their transport activities to some form of metabolic energy. Can the authors be more clear on whether they can say one way or another? Whatever the conclusion – or ambiguity – this point should be explicitly discussed.

**Author Rebuttals to Initial Comments:**

**Referees' comments:**

**Referee #1 (Remarks to the Author):**

Bacteria synthesize precursors of peptidoglycan cell wall and other envelope glycopolymers in the cytoplasm bound to the lipid carrier undecaprenyl-phosphate (C55-P). These precursors are flipped across the cytoplasmic membrane and, once the precursor moiety is used, the C55-P carrier is transported back across the membrane so that C55-P can be recycled. This recycling is an essential process (as inferred by the loss of viability caused by the antibiotic bacitracin) that occurs through an unknown mechanism. This study by Sit et al. focuses on identifying candidate C55-P transporters.

The authors propose that members of the DUF368 and DedA protein families function as C55-P transporters. The authors showed that Vibrio cholerae and Staphylococcus aureus mutants lacking members of the DUF368 family exhibit growth and peptidoglycan defects in alkaline pH conditions. In Vibrio, these defects were suppressed by lowering the Na+ concentration in the media and by suppressors that presumably have reduced Na+ import into the cytoplasm. The authors also showed synthetic interactions between genes encoding both putative transporters (DUF368 and DedA). Furthermore, in Vibrio, the single dedA mutant cannot grow in NaCl-free media and the synthetic lethality between DUF368 and dedA is suppressed by acidic pH. Consequently, the authors propose that DUF368 proteins, DedA, and a yet-to-be-identified protein(s) are C55-P transporters. They also conclude that the function of these transporters is determined by environmental conditions. Specifically, Sit et al. propose that DUF368 requires sodium ions to function, while DedA and the yet-to-be-identified protein(s) require protons.

The manuscript deals with a very important question. The data are of high quality, the work is extensive, and many of the main conclusions are supported by the data with the notable exception that the authors do not show direct evidence that these proteins are C55-P transporters. The authors establish a connection between DUF368 and cell wall defects, in agreement with the proposed function. However, a direct link between these proteins and C55-P transport is missing. This is an important issue given that previous studies have reported that the loss of DedA proteins results in pleiotropic defects.

We thank the referee for their detailed read of our manuscript and their positive comments. The identification of candidate C55-P translocases and investigation of their conditionality is a major step forward in our understanding of the biogenesis of cell surface glycopolymers including peptidoglycan. We agree that a direct link between DUF368 and DedA family proteins and C55-P translocation is not established by our work. Consistent with the title of our manuscript, we consider these proteins candidates, and future biochemical and structural studies will be necessary to definitively show transport functions. These experiments represent a new area of study that is outside of the scope of our current work, as both Referee #2 and #3 noted. We believe that the body of evidence presented in our revised manuscript, which now includes direct quantification of C55-P accumulation in DUF368-deficient cells, thus linking DUF368 proteins to C55-P homeostasis, is sufficient to propose DUF368 and DedA protein family members as C55-P translocases. However, we recognize we may have overstated some of our conclusions and have appropriately revised the text to avoid any claims that were not fully substantiated.

The referee raises a valid point about the pleiotropic phenotypes of DedA family proteins. This is certainly true – *dedA* mutants in various bacteria have growth and cell division defects, altered sensitivity to antimicrobials, and cell surface alterations[1]. While one interpretation is that DedA proteins play diverse roles in membrane homeostasis, these phenotypes can also be viewed through the lens of disrupted C55-P recycling. For example, the colistin sensitivity phenotype of *Burkholderia thailandensis* DedA mutants is dependent on the Ara4N modification of LPS, which is itself dependent on

C55-P recycling[2]. Furthermore, DedA family mutants in *B. thailandensis* and *E. coli* have been reported to be alkaline-sensitive[3,4], findings which are consistent with our model of conditional redundancy as both of these organisms lack a predicted DUF368 homologue (i.e., an alkaline-specialized C55-P translocase).

Finally, we note that our work follows a similar track to the discovery of the lipid II flippase MurJ. The identity of the lipid II flippase was not known for decades, until a 2008 bioinformatic study first proposed that MurJ may fulfill this function[5]. A subsequent landmark study used a phenotypic *in vivo* activity assay to provide strong, <u>but ultimately indirect</u> evidence for this idea[6]. This finding has been widely accepted by the field, even though MurJ-lipid II *in vitro* binding was not demonstrated for another five years[7], and neither *in vitro* reconstitution of transport nor a lipid II-bound MurJ structure has been reported to date. The energetic coupling of MurJ is also not known, although it is thought that lipid II flipping is dependent on membrane potential[8]. Thus, even though our study does not definitively link DUF368/DedA proteins with C55-P translocation, we believe our work opens the doors to what will be an immensely fruitful area of study spanning multiple fields, including genetics, cell biology, and biochemistry.

Specific major issues:
1) The study does not present evidence showing a direct link between C55-P and these putative transporters. Are C55-P levels changed in the mutants because it cannot be recycled? Can the phenotypes associated with the loss of these transporters be rescued by increasing C55-P production? Do the proteins bind C55-P? The loss of these proteins could be affecting peptidoglycan synthesis indirectly. For example, is the loss of these proteins leading to peptidoglycan defects because their loss alters membrane potential, which can then affect peptidoglycan synthesis in other ways (such as affecting lipid II transport and/or C55-P transport by the actual transporter)? Answering some of these questions would provide stronger support of their model.

As mentioned above, the reviewer is correct that our study does not directly show that DUF368/DedA proteins are C55-P transporters; however, our demonstration of C55-P accumulation in DUF368-deficient cells now provides a direct link between C55-P homeostasis and DUF368 proteins. We address the individual questions raised separately below:

<u>Are C55-P levels changed in the mutants because it cannot be recycled?</u>
This is a key question and we would expect that disruption to C55-P recycling leads to changes in undecaprenyl levels, likely an accumulation due to increased synthesis. To directly address this issue, we developed an HPLC-based assay to quantify extracted *S. aureus* membrane lipids. Using C55-OH and C55-P standards, we were able to detect peaks corresponding to these undecaprenyl moieties from WT and mutant *S. aureus* (Figure 3). Treatment of *S. aureus* with amphomycin led to a marked increase in both C55-OH and C55-P, suggesting that disruption of C55-P internalization leads to total undecaprenyl accumulation (Fig. 3C, bottom trace). This is consistent with the observation that C55-OH is a major lipid in Gram-positive bacteria and evidence that

C55-OH can be directly synthesized in Gram-positives and converted into C55-P by undecaprenol kinase[9,10]. Critically, C55-OH and C55-P levels in the Δ*SAOUHSC_0846 S. aureus* mutant phenocopied amphomycin treatment at alkaline but not neutral pH (Fig. 3D-G). We also quantified C55-P levels in *V. cholerae* and found a similar alkaline-dependent increase in C55-P in Δ*vca0040* cells (Extended Data Figure 5E, F). As the findings in *S. aureus* report on both C55-P and its precursor, these data suggest that there is a compensatory increase in C55-P synthesis when recycling is impaired (i.e., the cell senses a lack of C55-P flux due to surface accumulation). **These data provide a direct link between DUF368 domains and C55-P homeostasis and are a key addition to the revised work, allowing use to refocus the manuscript on these findings.**

Can the phenotypes associated with the loss of these transporters be rescued by increasing C55-P production?

This is an interesting question. In the co-submitted report from Roney and Rudner, deletion of the *uppS* or *ispH* genes had a minor synthetic deleterious effect on *Bacillus subtilis* lacking a DedA protein. These genes participate in the primary C55-P synthesis pathway, which assembles C55-PP that is subsequently dephosphorylated by an unknown mechanism to produce C55-P. Our C55 quantification described above suggests that an alternate synthetic pathway, mobilization of the C55-OH pool to produce more C55-P, may also contribute (Figure 3). Therefore, we believe that manipulation of various C55-P synthetic pathways may be more complicated than anticipated and constitutes an area for independent future study.

Is the loss of these proteins leading to peptidoglycan defects because their loss alters membrane potential?

To determine whether membrane potential is disrupted in the Δ*SAOUHSC_00846 S. aureus* mutant, we used a flow cytometry-based fluorescence staining assay with the voltage-sensitive dye DiOC2 (Fig. R1). This analysis clearly demonstrated that membrane potential is intact in the mutant and similar to the WT.

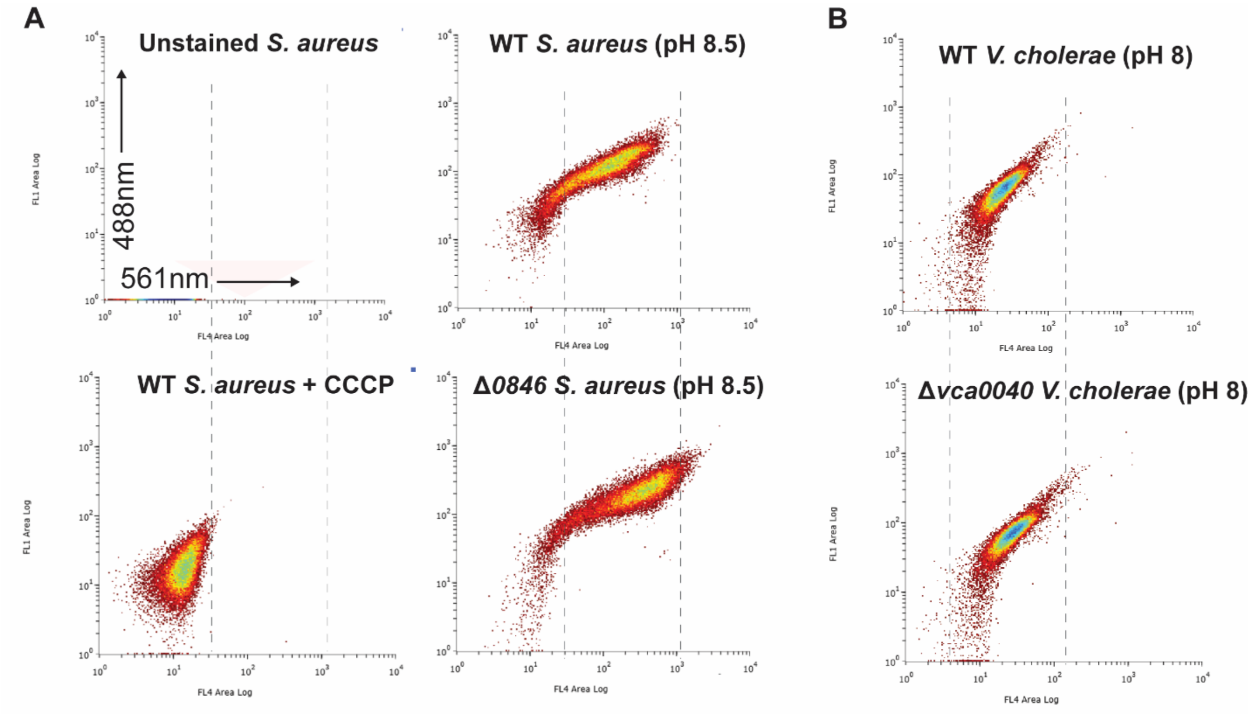


**Figure R1. Δ*0846 S. aureus* and Δ*vca0040 V. cholerae* do not have deficient membrane potential.** Cells were cultured in TSB-bicine at pH 8.5 (*S. aureus*) or LB at pH 8 (*V. cholerae)* until mid-exponential phase, labeled with 30μM 3,4=-diethyloxacarbocyanine iodide (DiOC2) and analyzed on a flow cytometer using 488nm and 561nm lasers. Carbonyl cyanide 3-chlorophenylhydrazone (CCCP; 5 uM) was used as a positive control for membrane depolarization. Representative plots are shown of 3 independent cultures for each condition.

2) The authors link these proteins to C55-P indirectly by using the antibiotic amphomycin. They show that the mutant is specifically sensitive to this drug, which binds C55-P. They also see an increase in fluorescence in S. aureus mutants lacking DUF368 when using fluorescently labeled amphomycin. What is the evidence showing that amphomycin is only present on the extracytoplasmic side of the membrane? In addition, the sensitivity and fluorescence results could also be explained by proposing that the DUF368 protein directly or indirectly affects the efflux of amphomycin. Can this alternative interpretation be ruled out?

There are both literature-based and experimental points that we believe argue against amphomycin labeling of the cytosolic face of the membrane:

1) From a biochemical standpoint, it is unlikely a bulky, amphipathic lipopeptide with a large polar surface such as amphomycin would be able to traverse the cell membrane, as concluded by others in the field[11]. There are no known uptake mechanisms for similar charged peptide antibiotics such as bacitracin.
2) Initial characterization of amphomycin and similar analogues showed this antibiotic does not affect membrane integrity unlike membrane-disrupting agents

such as daptomycin[12–14], suggesting that from the standpoint of its mechanism of action, amphomycin is unlikely to promote its own entry into the cell.

3) With respect to the influence of DUF368 and DedA proteins on antibiotic efflux, we think this is an unlikely explanation since neither protein family bears sequence resemblance to known efflux pumps, and because we only observed alkaline potentiation for the functionally related antibiotics amphomycin, tunicamycin, and bacitracin (and not the more general effect that would be expected if efflux was altered).

To further support this idea, we tested whether membrane integrity was compromised in our system, we added propidium iodide (PI) staining to the ampho-FL staining assay and only quantified cells that were PI-. This experimental change did not alter the magnitude of differential staining, and the level of PI staining was similar in WT and mutant cells, suggesting membrane permeability does not explain our results (Fig. R2).

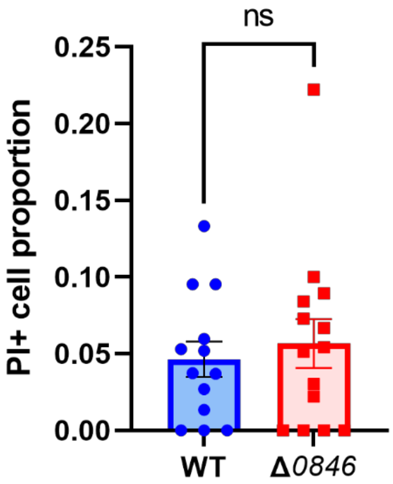


**Figure R2. PI-positive cells in *S. aureus* grown in TSB pH 8.5 from ampho-FL labeling experiments.** Cells were stained as described in the manuscript and imaged on an agarose pad containing 1µg/mL PI. Each symbol represents one field of view with at least 10 bacteria. Fields of view from three independent staining experiments were combined for analysis. ns: $p > 0.05$ by Mann-Whitney U test.

3) The authors show synthetic genetic interactions between genes encoding DUF368 and DedA proteins. Are the double mutant cells exhibiting cell wall defects? Is that why they die? The authors should show more detailed characterization of the synthetic phenotypes. At the very least, they should show growth curves and microscopy data linking peptidoglycan defects to the inability to grow.

Direct imaging of double mutant cells revealed a high frequency of aberrant cell shapes, suggesting that these cells have lost cell wall integrity and cannot maintain envelope homeostasis, likely leading to their death (Fig. R3).

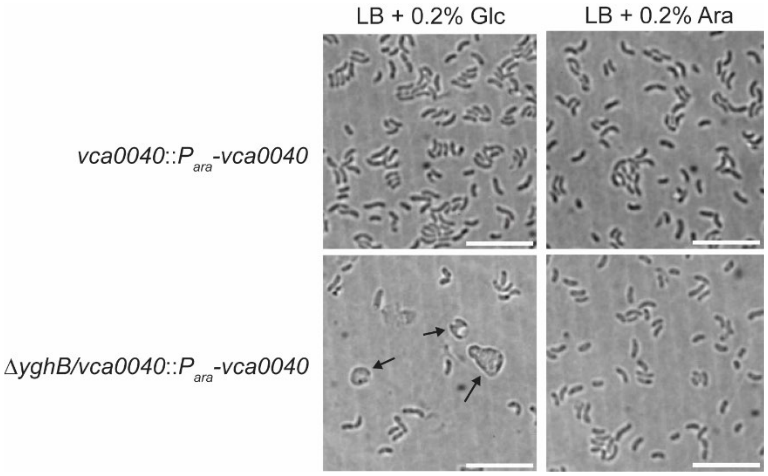


**Figure R3. DIC imaging of *vca0040*-depleted WT (top) or Δ*yghB* (bottom) *V. cholerae.*** Cells were streaked out on the indicated agar plates and incubated at 37°C overnight. Colonies were scraped and transferred to an agarose pad for imaging. Arrows highlight cells with obvious shape defects in the double mutant. Scale bar, 10μm.

4) Can the inability of the yghB (dedA paralog) single mutant to grow in the absence of NaCl be rescued by low pH? According to their model, suppression would be expected.

We have performed this experiment and found that as expected, the Δ*yghB V. cholerae* mutant was rescued by acidic conditions when grown in the absence of NaCl (Fig. R4). This finding is consistent with the low pH rescue of the double mutant and strengthens the possibility of a third transporter or spontaneous scrambling of protonated C55-P (as raised by Referee #3 and now included in the text (Lines 220-221)).

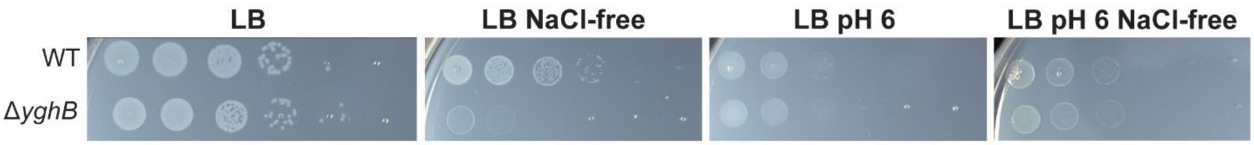


**Figure R4. Growth of Δ*yghB V. cholerae* on LB plates without NaCl at different pH.** Images are representative of three independent plated cultures.

5) In V. parahaemolyticus, the DUF368 protein VPA1624 is essential even at neutral pH (Figure S10B). Does this mean that the DedA homolog YghB does not perform the same function? Or is yghB not expressed under those conditions? Or is there another protein transporting C55-P? This finding seems to contradict their model and requires further experimentation.

We included this data to highlight the potential diversity in conditional contributions of DUF368 in different species. That is, while the general model that DUF368 proteins enable alkaline adaptation seems to hold true in *V. parahaemolyticus,* species-specific

networks and factors may alter the pH setpoint of such contributions. For example, *V. parahaemolyticus* may lack additional pH adaptation factors that are present in *V. cholerae,* or may simply rely on DUF368 activity at a wider pH range than *V. cholerae* because of species-specific low DedA protein activity. We have elected to keep this data in the manuscript to demonstrate this point and have clarified the relevant text (Lines 308-311).

To support this idea, we were able to rescue the growth of the DUF368 deletion in *V. parahaemolyticus* by growing the strain at pH 6, conditions where DUF368 reliance is likely lowered and reliance on DedA family proteins is enhanced. This is consistent with the idea that C55-P translocase activity in *V. parahaemolyticus* is still conditional, but at a shifted spectrum compared to that of *V. cholerae.* This is now shown in Extended Data Figure 10.

Minor points:

1) Figure 1H, Figure 2C, G, and H should include complementation data.

We have performed these complementation experiments and now include the data in the relevant figures.

2) Please include a scale bar in Fig. 2G. Also, it would be nice to compare data for the ΔSAOUHSC_00846 S. aureus mutant at acidic and neutral pH for fluorescein-conjugated amphomycin (ampho-FL).

We have repeated our amphomycin labeling experiments with complementation (Fig. 3F, G) and at pH 6, 7 and 8.5 (Extended Data Fig. 5G, H) and added this data to the manuscript. There was no difference in labeling intensity between the strains at acidic or neutral pH, consistent with our model of alkaline-restricted DUF368 C55-P translocase activity.

3) Fig. 3D and F: What are the dilutions shown in the spot assays?
The dilutions are ten-fold dilutions of an $OD_{600}$ 0.1 culture. This has been labeled in the revised figure.

4) Table S4: At what pH were the MIC assays done for the wildtype and the Δvca0040 mutant of V. cholerae? They should have been done at alkaline pH to be relevant.

The initial dataset was acquired at neutral pH as we had not uncovered the alkaline pH phenotype at that time. We have now examined selected antibiotics at alkaline pH for Δ*vca0040 V. cholerae* (Supplementary Table 5). We did not observe striking MIC differences at elevated pH, consistent with the idea that most PG-targeting antibiotics do not readily permeate the outer membrane and underscoring the importance of performing targeted studies in a more widely susceptible species such as *S. aureus*.

5) Line 163-162: “Na+-free, but not K+-free conditions suppressed the alkaline growth

and PG defects of Δvca0040 V. cholerae (Figs. 2A, B, 3A, Extended Data Figs. 5C, 6I, J).” This statement is misleading. Figs. 2A and 3A show that, but Fig. S6J shows that the mutant grows better at 5, 10, and 50 mM Na+ salt than at 0 mM Na+ (it does worse at 100 and 200 mM).

The referee is correct that low amounts of $Na^+$ stimulate mutant growth. This is likely due to the fact that *V. cholerae* is a halophile and requires some available $Na^+$ for optimal growth in ways that do not necessarily relate to VCA0040 function[15]. For example, aerobic *V. cholerae* respiration requires $Na^+$ translocation by the NQR oxidoreductase[16]. At low [$Na^+$], the positive effects of sodium on *V. cholerae* growth promote growth of the Δ*vca0040* mutant. However, once these positive effects plateau, the negative consequences of high [$Na^+$] in the mutant, which may be manifold, occur. To avoid any confusion, we have reworded this sentence to “Indeed, increasing [Na+], but not [K+] in the media induced alkaline growth and PG defects in Δvca0040 V. cholerae (Figs. 2B, C, Extended Data Figs. 7I-K)” (Lines 242-244).

6) It might be better to test salt dependency of both transporters by using various Na+ concentrations (like in Extended Data Figure 6J) in a Δvca0040 strain complemented by either VCA0040 or YghB.

We have now performed this experiment, and the results are consistent with our idea that VCA0040 and YghB operate via distinct mechanisms, since complementation with *vca0040* completely rescues the phenotype, whereas expression of *yghB* rescues most of the phenotype, but not the Na+ sensitivity of the mutant strain (Fig. R5).

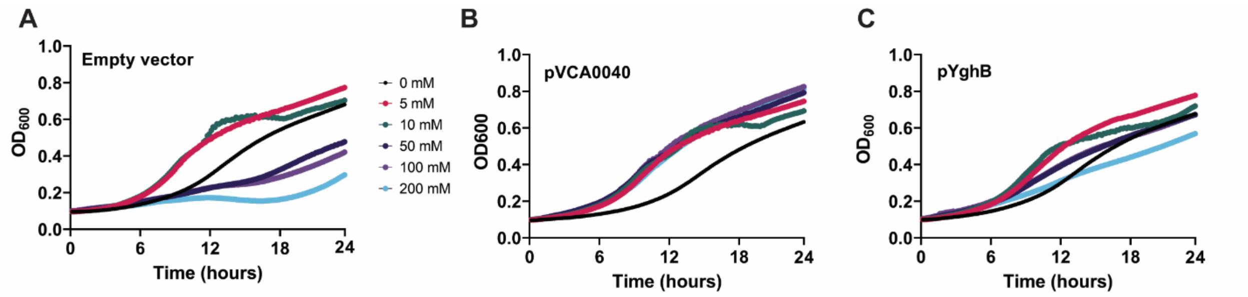


**Figure R5. Growth of Δ*vca0040 V. cholerae* expressing either *vca0040* or *yghB* with differing [$Na^+$].** Cells were grown in M63 medium buffered with KOH to pH 8. Cells were transformed with empty vector (A) or pBAD18 carrying *vca0040* (B) or *yghB* (C). Growth curves were performed in the presence of 0.2% arabinose and the average of 8 technical replicates is shown.

__Referee #2 (Remarks to the Author):__

In their wide ranging and comprehensive work on an unknown, conserved protein in the DUF368 family, essential for V. cholera pathogenesis, Waldor and colleagues establish that it likely performs Und-P recycling, a critical step in the generation of envelope glycopolymers. Previously, no protein was associated with performing this function.

Their comprehensive analysis provides rigorous in vivo evidence that this protein does perform Und-P recycling, and lacks only in vitro analysis (which is likely beyond the scope of this work) to fully establish its role. In the course of their studies, this group also showed that knocking-out a homologous gene in the gram positive organism S. aureus causes very similar phenotypes, establishing that the function spans the gram positive/negative divide. Examining DUF368 function in S. aureus also allowed them to provide further evidence of its role by showing that its phenotypes are consistent and synergistic with those of amphomycin, known to
prevent recycling, and that loss of this protein confers enhanced sensitivity to amphomycin. Additionally, the group examined its regulation, and function in varying contexts including pathogenesis, indicating multifaceted regulation and redundancy, as expected from a critical cellular function. Finally, using several complementaty transposon screens, they identified and validated a synthetic lethal interaction with a DedA protein, thereby identifying a second family of proteins to carry out this function. This already strong paper would have greater impact with the addition of one experiment to generalize their findings.

We thank the reviewer for their positive remarks and constructive assessment of our work.

1. Solidifying the S. aureus phenotypes. The authors already show that the DUF368 protein in S. aureus functions similarly to the V. cholera one, and that overexpression of 2 S. aueus DedA proteins rescued the DUF368 deletion phenotype. However, they do not report whether deletion of all DedA proteins in addition to DUF368 is possible and what the phenotypes of the triple mutant are. This is important to their framing of the broader scope of the ms,--showing that at least under some conditions, the proteins they identify have synthetic lethal phenotypes, consistent with loss of Und-P recycling upon loss of these functions.

We thank the referee for this excellent suggestion to use additional *S. aureus* mutants to strengthen our study. Initially, we created the single *S. aureus* mutant strains necessary for combinatorial mutant generation. Interestingly, closer examination of these additional single mutant strains led to new data that more strongly supports a role for DedA proteins as conditional C55-P translocases. In particular, MIC profiling of the single *S. aureus* mutants revealed a moderate (4x) sensitivity to amphomycin but not other antibiotics in the *SAOUHSC_02816::kan* (Δ*2816*) strain (Fig. 4F, Supplementary Table 5). Critically, this phenotype was only revealed at pH 6, which is consistent with the idea that DedA proteins have maximal functions at different pH setpoints than DUF368. This result also solidifies the overall conclusion that DedA family members are involved in C55-P transport.

We next created combinatorial double mutant strains, including Δ*0846/0901* and Δ*2816/0901.* However, their phenotypes did not differ from the Δ*0846* and Δ*2816* single mutants, respectively. We were unsuccessful at multiple attempts at creating the Δ*0846/2816* double mutant but the work of the accompanying paper from Roney and Rudner demonstrated that this mutant had a severe growth defect, consistent with a

requirement for a functional C55-P translocase. It is also our understanding that in their revised work, the triple Δ*0846/2816/0901* mutant was non-viable. Moreover, our existing dataset already illustrates the genetic interactions of DUF368 and DedA proteins in microbes that have both types of translocase candidates. We demonstrated in *V. cholerae* both synthetic lethality of *vca0040* and *yghB*, as well as the capacity of *yghB* to rescue the alkaline defect of *vca0040* when overexpressed. In *S. aureus,* the same overexpression-based rescue of DUF368 mutants by DedA proteins was observed. These data in aggregate reinforce the idea of synthetic interactions between DUF368-containing and DedA family proteins and provide strong evidence for conditional contributions of DedA family members to robust C55-P recycling.

2. It seems important to highlight bacitracin phenotypes, as they are immediately upstream of the step impacted by these new enzymes, and phenotypes are consistent with their model.

We now explicitly refer to the bacitracin phenotype in Lines 145-146.

Minor

-L26 “PG biosynthesis and structure are responsive to shifting environmental conditions…” There are 4 refs here, all published in 2019-2020, all with the same first and last author. Is this really the extent of the field?

We have added additional selected historical references on how environmental conditions shape cell wall structure and physiology (References #2-6 in the revised manuscript)

-L120. I’m not sure that this is the best citation for the MoA of amphomycin.

We have added additional references that support the mechanism of action of amphomycin to this line (References #21-23 in the revised manuscript).

**Referee #3 (Remarks to the Author):**

This is an important paper, presenting a significant advance.

We thank the reviewer for the positive assessment of our work.

Comments:

Lines 56-58: This sentence should be re-worked to include information on why BacA is not a credible C55-P translocase candidate.

Given the field’s focus on deciphering BacA’s phosphatase activity rather than a potential C55-P translocase activity, we are not aware of information on why BacA is not a credible candidate for this function. Rather, it is our understanding that the recently

presented structures of BacA[17–19] suggested this protein has structural features reminiscent of membrane transporters, thus opening the door to hypotheses that attribute translocase activity to BacA. As our work did not involve BacA, we remained intentionally agnostic to its role. Given that DUF368 and DedA double mutants are conditionally viable in *V. cholerae*, a third translocase may exist, and BacA is a reasonable candidate for that activity.

To assess whether BacA may be capable of performing C55-P translocation, we attempted overexpression-based rescue of *V. cholerae Δvca0040* bacteria with *V. cholerae uppP* (*bacA*). We did not observe alkaline rescue, suggesting that BacA is not a C55-P translocase (Fig. R6). However, in our view, these data are also consistent with a narrow conditional or tertiary contribution of BacA to translocation, which is a question for future study. We have reworded this sentence to "While preliminary structural studies have proposed that UppP/BacA may also function as a C55-P translocase, the protein(s) responsible for C55-P internalization have not yet been identified" (Lines 54-56)

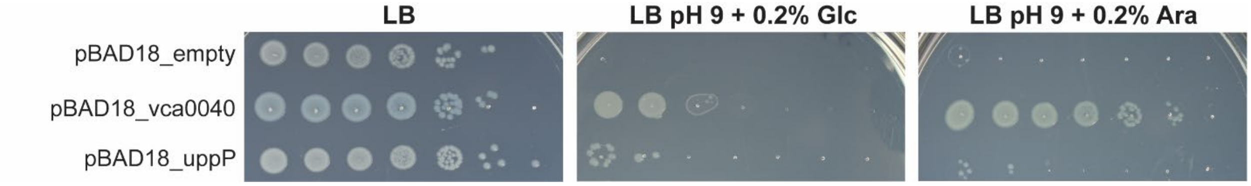


**Figure R6. *uppP* overexpression does not rescue Δ*vca0040 V. cholerae* at alkaline pH.** Drip dilutions were performed as described in the Methods section of the manuscript.

Lines 127-129: The ampho-FL labeling (Fig 2G, H) was done at alkaline pH 9 – the extent of labeling would be expected to be pH dependent based on the other phenotypes reported. This should be explicitly shown by doing the staining at acidic pH.

We have performed the ampho-FL staining at neutral and acidic pH and indeed show that it is pH-dependent as would be expected, with no difference between WT and Δ*0846 S. aureus* in non-alkaline conditions (Extended Data Fig. 5G, H).

Lines 138-139: The docking data are of mixed value. Docking is performed under highly non-natural conditions, and demonstration of a lock and key arrangement of VCA0040 with C55-P seems to run counter to a transport function. How would such a perfectly fitted lipid be transported?

This is a valid point. As the in-depth biochemical and structural evidence required to demonstrate C55-P binding parameters are beyond the scope of the current study, we have removed this speculative modeling from the revised manuscript.

Line 179: Does the yghB mutant exhibit a growth defect at pH>8 in sodium replete conditions?

We have performed this experiment and Δ*yghB V. cholerae* do not exhibit a growth defect at alkaline pH in $Na^+$-replete conditions, as expected since VCA0040 should be most active in these conditions (Fig. R7).

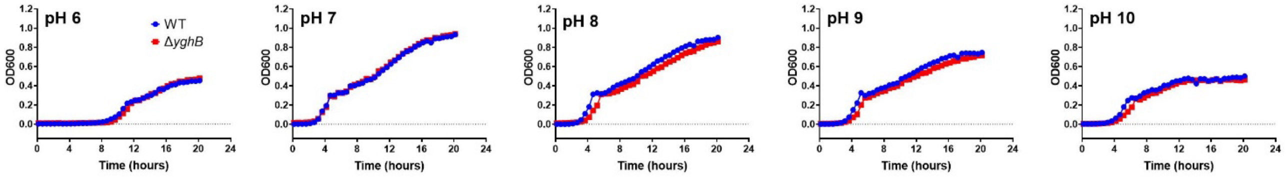


**Figure R7. Growth of Δ*yghB V. cholerae* at differing pH in $Na^+$-replete media.** The mean $OD_{600}$ of four technical replicates is shown.

Line 191: The eukaryotic DedA proteins TMEM41B and VMP1 have been shown to scramble glycerophospholipids – there are no data to indicate lipid binding.

This phrase has been reworded to: "are associated with lipid transport" (Line 204) (see also response below about scramblase activity).

Line 193: Because the double mutant (vca0040, yghB) grows under acidic conditions, the authors conclude that there must be at least one other protein responsible for C55-P translocation. This is reasonable. However, protonation of the phosphate group under suitably acidic conditions would promote spontaneous scrambling of C55-P, akin to that reported for phosphatidic acid (PMID1993189). This possibility should be discussed with appropriate citation.

The reviewer raises a valid point. Protonation of the phosphate in C55-P in acidic conditions could allow spontaneous scrambling. However, the lowest pKa of C55-P is estimated to be ~1.79 (http://pseudomonas.umaryland.edu/PAMDB?MetID=PAMDB001054), suggesting that even if the first protonation occurs at a physiological pH, the second protonation is unlikely to occur in a normal cellular environment. This would suggest that C55-P is unlikely to be neutrally charged *in vivo*. The activation energy to flip a charged lipid without a dedicated transporter would be quite high. The formula from the paper provided by the reviewer suggests that spontaneous flipping at 37°C at pH 6 would have a half-life of 21 hours, which is likely not physiologically relevant. Nonetheless, as we cannot exclude this possibility, we have included it in the revised text at Lines 220-221 and 290-292.

Related to line 193: Under alkaline conditions, the vca0040 mutant grows in low sodium but the yghB mutant does not. And vice versa. The model in Extended Data Figure 6E suggests the differential roles of the Sec mutant suppressors, with the implication that Na- or proton-motive force is used differentially to support one or other of the C55-P translocases – the effect could be direct or indirect. Much is made of the idea that environmental conditions control which translocase is relevant, but what would be the

evolutionary advantage in this system? One environmentally insensitive translocase should do the trick. There is no apparent redundancy in the Lipid II translocation machinery, so why would the recycling of C55-P require redundancy? It would be worth discussing these points.

The reviewer raises interesting questions. Although one environment-independent translocase could in theory fit the needs of most microbes, we think it is likely that environment-specific selection and usage of translocases serves to optimize and broaden cellular fitness. The biological role of C55-P is not restricted to PG biosynthesis, as is the case for Lipid II. C55-linked carriers participate in the biosynthesis of an array of cell surface glycopolymers, including PG, LPS, WTA, and the archaeal S-layer. This near-universal requirement for C55-P recycling in cell surface maintenance suggests that redundancy would be a valuable mechanism to avoid targeting by antibiotics and/or to enable environment-specific maximization of fitness. Even within PG biosynthesis, functional redundancy exists in the diverse array of enzymes that shape the cell wall with differing environmental sensitivities[20]. Additionally, the enzymatic step performed by MurJ may encode some redundancy, as alternate Lipid II flippases have been proposed[21].

The idea of functional redundancy does not necessarily rely on differential energetic inputs, although it is our preferred hypothesis. Our data are also consistent with differential gene expression/post-transcriptional activation of different translocases in certain environments. These mechanisms may coordinate with other pathways that regulate C55-P synthesis and degradation to fine-tune conditional microbial cell surface adaptation. Overall, we prefer the energetic explanation because it is an integrative signal from the environment to which microbes are already known to sense and respond. Since microbes vary vastly in their energetic reliance on Na/H gradients[22], it is logical that $Na^+$ and $H^+$ powered core transporters could exist. Retention of multiple translocases would thus optimize microbial adaptive potency to harsh new environments. To accommodate alternative models and explanations, we have introduced a more nuanced version of this argument in the discussion.

The paper describes comprehensive genetics-based analyses but lacks a direct test of the C55-P transport activity of the VCA0040 and YghB proteins. While such a test is beyond the scope of the paper, the authors should point out that a definitive conclusion about the role/activity of the candidate translocases requires a biochemical test using purified proteins.

We agree that a direct biochemical test of C55-P transport will be critical for definitively assigning translocase function to VCA0040 and YghB and have added a sentence addressing this specific future direction to the discussion (Lines 281-284).

The TMEM41B and VMP1 DedA proteins in eukaryotes have been shown to be glycerophospholipid scramblases. The implication in this paper is that the candidate C55-P transporters couple their transport activities to some form of metabolic energy.

Can the authors be more clear on whether they can say one way or another? Whatever the conclusion – or ambiguity – this point should be explicitly discussed.

The reviewer is correct that our data do not definitively show whether transport is directional and coupled to metabolic energy. This was an inference from our observation that translocase contributions were pH-dependent, since a key function of pH is to alter the energetic state of the cell, and in some microbes like *V. cholerae*, drive differential energy motive force usage. Given that *V. cholerae* is recognized to leverage both SMF and PMF-driven processes to support fitness, we reasoned that a similar divergence may account for differing contributions of DUF368 and DedA proteins to C55-P transport. Ultimately, however, we acknowledge this is a conjecture and not a conclusion and have appropriately scaled back our claims and model (Fig. 5H).

**References**


1. Doerrler, W. T., Sikdar, R., Kumar, S. & Boughner, L. A. New functions for the ancient DedA membrane protein family. *J. Bacteriol.* **195**, 3–11 (2013).
2. Panta, P. R. & Doerrler, W. T. A Burkholderia thailandensis DedA Family Membrane Protein Is Required for Proton Motive Force Dependent Lipid A Modification. *Front. Microbiol.* **11**, 618389 (2020).
3. Panta, P. R. & Doerrler, W. T. A link between pH homeostasis and colistin resistance in bacteria. *Sci. Rep.* **11**, 13230 (2021).
4. Kumar, S. & Doerrler, W. T. Escherichia coli YqjA, a member of the conserved DedA/Tvp38 membrane protein family, is a putative osmosensing transporter required for growth at alkaline pH. *J. Bacteriol.* **197**, 2292–2300 (2015).
5. Ruiz, N. Bioinformatics identification of MurJ (MviN) as the peptidoglycan lipid II flippase in Escherichia coli. *Proc. Natl. Acad. Sci. U. S. A.* **105**, 15553–7 (2008).
6. Sham, L.-T. *et al.* Bacterial cell wall. MurJ is the flippase of lipid-linked precursors for peptidoglycan biogenesis. *Science* **345**, 220–2 (2014).
7. Bolla, J. R. *et al.* Direct observation of the influence of cardiolipin and antibiotics on lipid II binding to MurJ. *Nat. Chem.* **10**, 363–371 (2018).
8. Rubino, F. A., Kumar, S., Ruiz, N., Walker, S. & Kahne, D. E. Membrane Potential Is Required for MurJ Function. *J. Am. Chem. Soc.* **140**, 4481–4484 (2018).
9. Kawakami, N. & Fujisaki, S. Undecaprenyl phosphate metabolism in Gram-negative and Gram-positive bacteria. *Biosci. Biotechnol. Biochem.* **82**, 940–946 (2018).
10. Baker, B. R., Ives, C. M., Bray, A., Caffrey, M. & Cochrane, S. A. Undecaprenol kinase: Function, mechanism and substrate specificity of a potential antibiotic target. *Eur. J. Med. Chem.* **210**, 113062 (2021).
11. Wang, Z., Koirala, B., Hernandez, Y., Zimmerman, M. & Brady, S. F. Bioinformatic prospecting and synthesis of a bifunctional lipopeptide antibiotic that evades resistance. *Science* **376**, 991–996 (2022).
12. Rubinchik, E. *et al.* Mechanism of action and limited cross-resistance of new lipopeptide MX-2401. *Antimicrob. Agents Chemother.* **55**, 2743–54 (2011).
13. Singh, M., Chang, J., Coffman, L. & Kim, S. J. Solid-state NMR characterization of amphomycin effects on peptidoglycan and wall teichoic acid biosyntheses in

Staphylococcus aureus. *Sci. Rep.* **6**, 1–9 (2016).
14. Schneider, T. *et al.* The lipopeptide antibiotic Friulimicin B inhibits cell wall biosynthesis through complex formation with bactoprenol phosphate. *Antimicrob. Agents Chemother.* **53**, 1610–8 (2009).
15. Häse, C. C. & Barquera, B. Role of sodium bioenergetics in Vibrio cholerae. *Biochim. Biophys. Acta - Bioenerg.* **1505**, 169–178 (2001).
16. Juárez, O. & Barquera, B. Insights into the mechanism of electron transfer and sodium translocation of the Na+-pumping NADH:quinone oxidoreductase. *Biochim. Biophys. Acta - Bioenerg.* **1817**, 1823–1832 (2012).
17. El Ghachi, M. *et al.* Crystal structure of undecaprenyl-pyrophosphate phosphatase and its role in peptidoglycan biosynthesis. *Nat. Commun.* **9**, 1078 (2018).
18. Workman, S. D., Worrall, L. J. & Strynadka, N. C. J. Crystal structure of an intramembranal phosphatase central to bacterial cell-wall peptidoglycan biosynthesis and lipid recycling. *Nat. Commun.* **9**, 1159 (2018).
19. Workman, S. D. & Strynadka, N. C. J. A Slippery Scaffold: Synthesis and Recycling of the Bacterial Cell Wall Carrier Lipid. *J. Mol. Biol.* **432**, 4964–4982 (2020).
20. Egan, A. J. F., Errington, J. & Vollmer, W. Regulation of peptidoglycan synthesis and remodelling. *Nat. Rev. Microbiol.* **18**, 446–460 (2020).
21. Meeske, A. J. *et al.* MurJ and a novel lipid II flippase are required for cell wall biogenesis in Bacillus subtilis. *Proc. Natl. Acad. Sci. U. S. A.* **112**, 6437–42 (2015).
22. Mulkidjanian, A. Y., Dibrov, P. & Galperin, M. Y. The past and present of sodium energetics: May the sodium-motive force be with you. *Biochim. Biophys. Acta - Bioenerg.* **1777**, 985–992 (2008).

**Reviewer Reports on the First Revision:**

Referees' comments:

Referee #1 (Remarks to the Author):

I would like to thank the authors for their thorough responses to the previous reviews. They have addressed my concerns satisfactorily, especially by establishing a more direct link between the DUF368-containing and DedA family of proteins to undecaprenyl-phosphate. It was never my expectation that a reconstitution was needed for this study. Congratulations on a beautiful and impactful piece of work.

Minor fix: Please check the supplemental tables names in their respective files and manuscript text. I think some of the numbers need to be fixed (e. g. Table S4 is labeled S3 in the file; S3 is labelled S6, and S6 is labelled S5).

Referee #2 (Remarks to the Author):

The authors do an excellent job of addressing our comments and those of the other reviewers.

Carol Gross

Referee #3 (Remarks to the Author):

The authors have responded carefully, including their thoughtful acknowledgement of the MurJ work that establishes a precedent for the style/logic/content of their own experiments.

## Author Rebuttals to First Revision:

### Referees' comments:

### Referee #1 (Remarks to the Author):

I would like to thank the authors for their thorough responses to the previous reviews. They have addressed my concerns satisfactorily, especially by establishing a more direct link between the DUF368-containing and DedA family of proteins to undecaprenyl-phosphate. It was never my expectation that a reconstitution was needed for this study. Congratulations on a beautiful and impactful piece of work.

Minor fix: Please check the supplemental tables names in their respective files and manuscript text. I think some of the numbers need to be fixed (e. g. Table S4 is labeled S3 in the file; S3 is labelled S6, and S6 is labelled S5).

We thank the referee for the positive comments and careful attention to detail – we double-checked all supplemental table files to ensure the within-file naming is consistent with the final table numbering.

### Referee #2 (Remarks to the Author):

The authors do an excellent job of addressing our comments and those of the other reviewers.

Carol Gross

Thank you, Dr. Gross, for your insightful comments on our manuscript.

### Referee #3 (Remarks to the Author):

The authors have responded carefully, including their thoughtful acknowledgement of the MurJ work that establishes a precedent for the style/logic/content of their own experiments.

We thank the referee for the positive assessment of our work.
